# Analysis of flow field characteristics of sand removal hydrocyclone applicable to solid fluidization exploitation of natural gas hydrate

**DOI:** 10.1371/journal.pone.0295147

**Published:** 2023-12-07

**Authors:** Na Wei, Yi Qiao, Shuanshi Fan, Meng Cai, Haitao Li, Shouwei Zhou, Jinzhou Zhao, Liehui Zhang, Richard Banks Coffin

**Affiliations:** 1 State Key Laboratory of Oil and Gas Reservoir Geology and Exploitation, Southwest Petroleum University, Chengdu, China; 2 State Key Laboratory of Natural Gas Hydrate, Beijing, China; 3 Daqing Oilfield of CNPC, Daqing, China; 4 Department of Physical and Environmental Science, Texas A&M University—Corpus Christi, Corpus Christi, TX, United States of America; NED University of Engineering and Technology, PAKISTAN

## Abstract

With the development of economy and society, the consumption of fossil energy is gradually increasing. In order to solve the current energy dilemma, Natural gas hydrate (NGH) is considered as an ideal alternative energy. At the same time, solid fluidization exploitation is an ideal method. However, in the process of that, sand and hydrate ore bodies enter the closed pipeline together, which will block the pipeline and increase the difficulty of exploitation. Therefore, the pre-separation of sand by hydrocyclone plays an important role in solid fluidization exploitation. In this study, the numerical simulation method was used to study the internal flow field characteristics of the hydrocyclone, and the effects of different flow rate, different flow ratio, different sand content and different particle diameter on the phase distribution were investigated. The results show that: at the same axial position, the increase of flow rate and sand content makes the sand phase more distributed at the edge of the flow field. Under the same working conditions, the sand gradually migrates to the center of the flow field with the increase of the axial distance. By calculation, it is obtained that under the optimum working condition of the flow rate is 4.83m^3^/h, the flow ratio is 20%, the sand content is 20%, and sand diameter is 80μm, the maximum *E*_s_ is 22.1% and the minimum is 86.1%. Finally, a comprehensive analysis of the hydrocyclone in this study shows that this hydrocyclone is only applicable to rough pre-separation of sand in the process of solid fluidization exploitation. Through the study of the internal flow field characteristics and phase distribution law of the hydrocyclone, this study provides a reference for the practical engineering application of sand phase pre-separation in the solid fluidization exploitation of NGH.

## 1. Introduction

With the development of economy and industry, the consumption of global fossil energy is gradually increasing. According to statistics, the total global energy consumption in 2018 was 14.301 billion tons of oil equivalent, of which oil accounted for 31%, coal accounted for 26%, and natural gas accounted for 23% [1, 2]. A large amount of fossil energy consumption makes social development fall into a dilemma of serious shortage of resources and rapid deterioration of ecological environment [3]. Therefore, finding an alternative clean energy has become the key to solve the current energy dilemma. Natural gas hydrate (NGH) is widely known for its abundant energy reserves, wide distribution, clean combustion and high energy density [4–6]. Natural gas hydrate is an ice-like and combustible clathrate crystalline compound formed by water molecules and light hydrocarbon gas molecules under low temperature and high pressure environment, so natural gas hydrate is also called “Combustible ice” [7]. Under standard conditions, 1m^3^ natural gas hydrate can release 164m^3^ natural gas [8, 9]. It is estimated that the current global natural gas hydrate reserves are about 2×10^16^m^3^, equivalent to 2×10^13^ tons of oil equivalent, about 40 times that of conventional natural gas reserves, and the content of organic carbon is twice that of the world’s proven fossil fuels [10–12].

Natural gas hydrates are mainly distributed in seabed sediments below 300m and in land permafrost layer at 200-2000m [13, 14]. Therefore, the reservoir-forming environment of low temperature and high pressure has become the main factor limiting the large-scale exploitation of gas hydrate. At present, the exploitation methods of natural gas hydrate mainly include depressurization method, thermal excitation method, chemical injection method and so on [15–18]. The depressurization method is to break the equilibrium pressure condition of the hydrate phase by lowering the reservoir pressure, so as to promote the decomposition of the hydrate [19]. The thermal excitation method is to promote the release of methane gas by injecting heat into the hydrate reservoir and breaking the temperature condition of hydrate phase equilibrium [20]. The chemical injection method is to inject natural gas hydrate inhibitors into hydrate reservoirs to decompose hydrates [21]. However, there are some disadvantages such as uncontrollable phase transition, high energy consumption, unstable gas production and high economic cost in the exploitation of natural gas hydrate by the above methods [22, 23].

In order to solve the problems existing in the exploitation of natural gas hydrate at present, Zhou put forward the solid state fluidization exploitation method of natural gas hydrate [24, 25]. The natural gas hydrate solid fluidization exploitation method is to use high-pressure jet or mechanical mining to break the solid hydrate ore body on the shallow surface of the seabed into fine particles, and then mix the broken hydrate ore body with seawater to form a hydrate slurry. The hydrate slurry is transported to the offshore platform through a closed pipeline for later separation treatment [26, 27]. The solid fluidization exploitation method changes the uncontrollable decomposition of gas hydrate into continuous controllable decomposition, which realizes the in-situ exploitation of natural gas hydrate and avoids catastrophic production accidents caused by hydrate decomposition in the process of exploitation [15, 28].

During the exploitation of natural gas hydrate by solid fluidization exploitation method, the hydrate slurry is transported from the seabed to the offshore platform in a closed pipeline [29]. With the change of temperature and pressure, the hydrate ore body gradually decomposes into three phases of gas, water and sand, and the flow changes from solid and liquid two-phase flow to gas, liquid and solid three-phase flow in the closed pipeline [30]. Therefore, the three-phase separation of gas, water and sand in the hydrate slurry is the key to realize the solid fluidization exploitation of natural gas hydrate. At present, the main methods used for separating hydrate slurry are gravity separation, chemical separation, and cyclone separation [31–33]. Among them, the cyclone separation method has attracted much attention because of its high efficiency, small size and high separation speed [34]. The hydrate slurry is injected into the hydrocyclone by the closed pipeline, and the mixture moves in a circle along the wall of the cyclone chamber. Due to the density difference of gas, water and sand, the centrifugal force difference is generated during the circular movement, thus the separation of different phases is realized.

At present, many experts and scholars have carried out extensive research on the application of three-phase hydrocyclone in the exploitation of natural gas hydrate. Qiu et al. [35] analyzed the impact of reservoir sand production on the exploitation of natural gas hydrate and designed an underground hydrocyclone based on this. At the same time, the structural parameters of the hydrocyclone were optimized by numerical simulation method, and the separation efficiency of the optimized cyclone separator was evaluated. Fang et al. [36] studied the response relationship of sand particle diameter, sand volume fraction and natural gas volume fraction with the gas collection efficiency and sand removal efficiency of hydrocyclone based on the small hydrocyclone with classical structure, providing a basis for the practical application of hydrocyclone in the solid fluidization exploitation of hydrate. Wei et al. [37] optimized the structural parameters of the traditional three-phase hydrocyclone by using the computational fluid dynamics method, analyzed the effect of various structural parameters on the separation efficiency of hydrate slurry in the hydrocyclone, and obtained the optimum combination of structural parameters of the hydrocyclone. Lin [38] designed an axial annulus in situ hydrocyclone desander (AAIHD), and explored the applicability of this hydrocyclone in solid fluidization exploitation of hydrate. Qiu et al. [39] used CFD method to study the influence of structural parameters of spiral inlet of hydrocyclone on sand removal and recovery of hydrate during the exploitation, which provided theoretical guidance for the engineering design of hydrocyclone applicable to in-situ separation of NGH. Duan et al. [40] put forward a method of using cyclone separation technology to consolidation breaking and sand removal of hydrate at the same time, and verified the feasibility and accuracy of the method. Tang et al. [41] discussed the application of cyclone separation technology in in-situ sand removal during solid fluidization exploitation of NGH. Qian et al. [42] studied the hydrocyclonic separation of NGH slurries combined with the solution of particles settling equation and plotted the grade efficiency curves of both NGH and sand particles. Chang et al. [43] proposed a hydrocyclone applicable to the exploitation of subsea natural gas hydrate. The effects of operational and structural parameters on the separation performance of the hydrocyclone were studied using a combination of numerical simulation and experiments, and the optimal ratio of structural parameters was obtained. However, so far, there are few studies on the internal flow field characteristics and phase distribution law of hydrocyclone used in the solid fluidization exploitation of natural gas hydrate.

In this paper, based on the axial-flow sand removal hydrocyclone which is applicable to solid fluidization exploitation of natural gas hydrate, the numerical simulation method is used to study the flow field distribution characteristics and phase distribution law in the hydrocyclone under the condition of water and sand two-phase and its applicability in solid fluidization exploitation of natural gas hydrate is evaluated. In order to ensure the universality and accuracy of the research, combined with the actual engineering situation, the effects of different flow rates, different flow ratios, different sand volume fraction and different sand particle diameter on the internal flow field characteristics and phase distribution of the hydrocyclone were studied. The sand discharge efficiency and water discharge efficiency of the hydrocyclone were calculated under different conditions. It provides some guiding significance for the practical engineering application of the hydrocyclone in the solid fluidization exploitation of natural gas hydrate.

## 2. Method

### 2.1 Physical model

**Fig 1:**
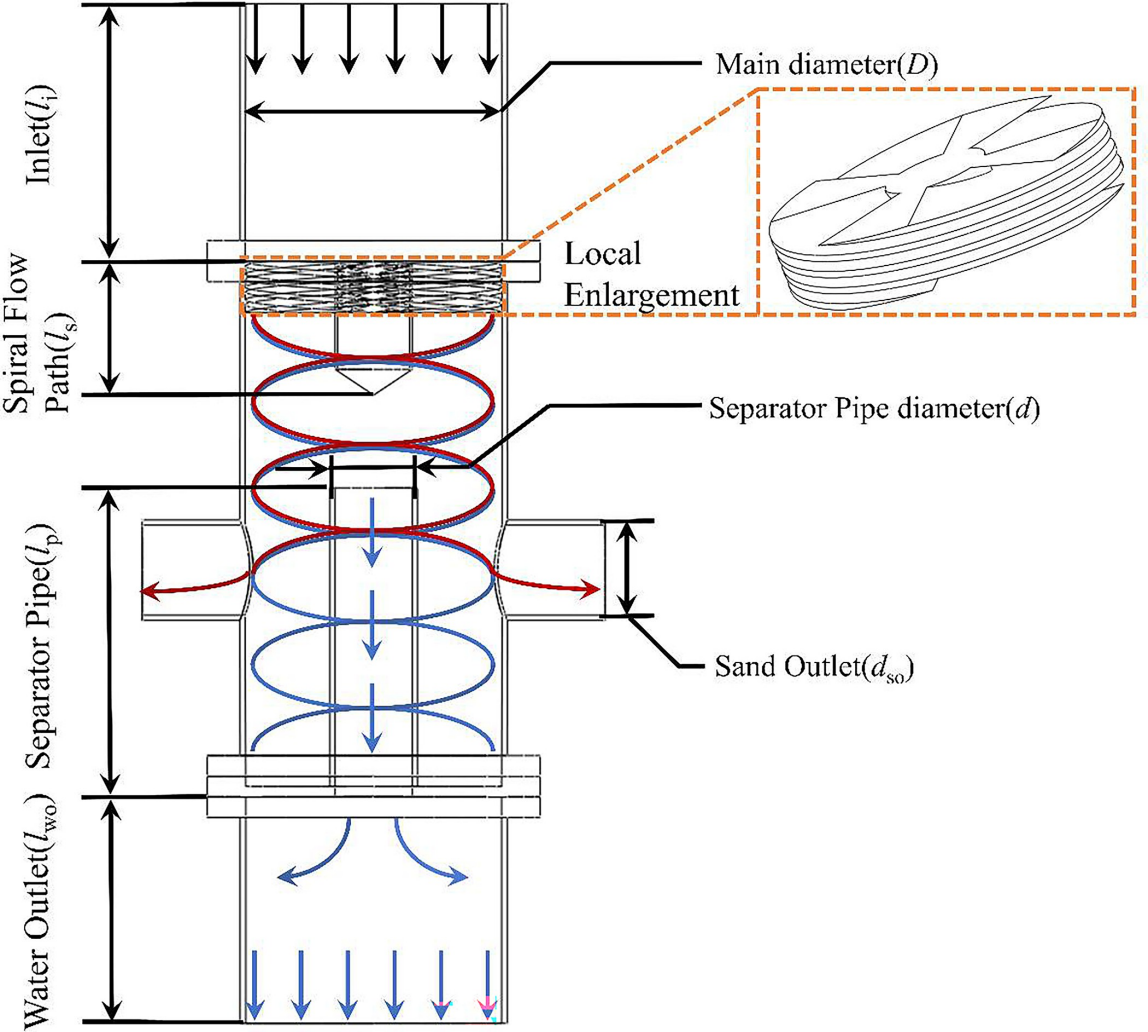
The structure of hydrocyclone for in-situ sand removal.

In this paper, based on the hydrocyclone proposed by Chang [43], which is applicable to in-situ separation of sand phase in solid fluidization exploitation of natural gas hydrate, the flow field characteristics and the distribution of different phase in the hydrocyclone are analyzed and the separation efficiency of the hydrocyclone is calculated. The structure of the hydrocyclone applicable to the in-situ separation of sand phase in the solid fluidization exploitation of natural gas hydrate is shown in Fig 1.

In the process of solid fluidization exploitation of natural gas hydrate, the shallow weakly cemented hydrate reservoir on the seafloor is broken into a hydrate ore body by mechanical crushing method or high pressure jet method, and then the hydrate orebody and sediment enter the closed pipeline with the sea water. Considering the narrow space in the actual natural gas hydrate mining project, an axial-flow hydrocyclone is used to initially separate the sand entering the pipeline at the exploitation site, and the separated sand is backfilled. As shown in Fig 1, the mixture of water and sediment enters the hydrocyclone from the inlet at an axial velocity, and after passing through the spiral flow path, part of the axial velocity of the mixture is transformed into a tangential velocity. At this time, the flow field inside the hydrocyclone become a swirling flow field. Due to the density difference between water and sand, a centrifugal force difference is generated in the swirling flow field. The density of the water phase is smaller than sand phase, and the centrifugal force generated in the circular motion is small, so the water phase is distributed at the axis of the hydrocyclone, and enters the separator pipe, ultimately discharged from the hydrocyclone through the water outlet. The density of the sand phase is relatively high, and centrifugal force generated in the circular motion is large, Therefore, the sand phase is distributed on the inner walls of the hydrocyclone, and finally discharged from the hydrocyclone through the sand outlet. Thus, the preliminary separation of sand phase in the process of solid fluidization exploitation of natural gas hydrate is completed.

In this study, a three-dimensional model was used to study the flow field characteristics and the distribution law of different phase in the in-situ sand removal hydrocyclone for solid fluidization exploitation of natural gas hydrate. In the numerical simulation of the swirling flow field, the three-dimensional model can more objectively and comprehensively reflect the differences of different physical fields and different phase in different directions, which plays a significant role in improving the accuracy of numerical simulation of the flow field in the hydrocyclone [44]. The dimensions of the hydrocyclone are shown in Table 1.

**Table 1:** Dimensions of the hydrocyclone.

Structure	Symbol	Value(m)
Main diameter	*D*	1×10^−1^
Separator Pipe diameter	*d*	2.5×10^−2^
Sand Outlet diameter	*d*_so_	3.5×10^−2^
Inlet length	*l*_i_	1×10^−1^
Spiral Flow Path length	*l*_s_	1×10^−1^
Water Outlet length	*l*_wo_	1×10^−1^
Separator Pipe length	*l*_p_	1.2×10^−1^

### 2.2 Mathematical model

#### 2.2.1 Governing equation

The fluid flow in a hydrocyclone can be regarded as a viscous incompressible fluid, which follows the basic governing equations as shown in Eqs (1)–(3) [45, 46]:

Continuity equation:

$$\frac{\partial }{\partial x_{j}}(\rho u_{j})=0$$ (1)

Momentum conservation equation:

$$\frac{\partial }{\partial x_{j}}(\rho u_{i}u_{j})=-\frac{\partial p}{\partial x}+\frac{\partial }{\partial x_{j}}(\mu \frac{\partial u_{i}}{\partial x_{j}})+(\rho -\rho_{a})g_{j}$$ (2)

Energy conservation equation:

$$\frac{\partial }{\partial x_{j}}(\rho u_{j}T)=\frac{1}{C_{p}}\frac{\partial }{\partial x_{j}}(k_{t}\frac{\partial T}{\partial x_{j}})+\frac{C_{pv}-C_{pa}}{C_{p}}[\frac{\partial }{\partial x_{j}}(\frac{\mu_{t}}{\sigma_{c}})\frac{\partial \omega }{\partial x_{i}}]\frac{\partial T}{\partial x_{j}}$$ (3)

#### 2.2.2 Turbulence modeling

In this study, the numerical simulation was performed via the commercial software Ansys’s Fluent 2020. Due to the fact that the internal flow field of the hydrocyclone is considered a strong vortex flow field, choosing the correct turbulence model has a significant impact on the accuracy of numerical simulation results. Among many turbulence models, the Reynolds Stress Model (RSM) takes into account continuity equation, momentum equation, transport equation and anisotropic turbulent shear equation at the same time, which is mainly applied to the numerical simulation of complex three-dimensional flow field considering Reynolds stress anisotropy. Therefore, the RSM is used to simulate the flow field in the hydrocyclone.

The Reynolds stress model is based on the average Reynolds number theory, and the governing equations are shown in Eqs (4)–(10) [47–50].

Reynolds stress transport equation:

$$\frac{\partial }{\partial t}(\rho \bar{u_{i}^{\prime }u_{j}^{\prime }})+\frac{\partial }{\partial x_{k}}(\rho u_{k}\bar{u_{i}^{\prime }u_{j}^{\prime }})=D_{T,ij}+P_{ij}+\Phi_{ij}+G_{ij}-\varepsilon_{ij}$$ (4)

Turbulent kinetic energy diffusion term equation:

$$D_{T,ij}=-\frac{\partial }{\partial x_{k}}(\rho \bar{u_{i}^{\prime }u_{j}^{\prime }u_{k}^{\prime }}+\bar{pu_{j}^{\prime }}\delta_{jk}-\mu \frac{\partial }{\partial x_{k}}\bar{u_{i}^{\prime }u_{j}^{\prime }})$$ (5)

Molecular viscous diffusion term equation:

$$D_{L,ij}=\frac{\partial }{\partial x_{k}}[\mu \frac{\partial }{\partial x_{k}}(\bar{u_{i}^{\prime }u_{j}^{\prime }})]$$ (6)

Shear stress equation:

$$P_{ij}=\rho (\bar{u_{i}^{\prime }u_{k}^{\prime }}\frac{\partial u_{j}}{\partial x_{k}}+\bar{u_{j}^{\prime }u_{k}^{\prime }}\frac{\partial u_{i}}{\partial x_{k}})$$ (7)

Buoyancy generation term equation:

$$G_{ij}=-\rho \beta (g_{i}\bar{u_{j}^{\prime }\theta }+g_{j}\bar{u_{i}^{\prime }\theta })=\beta \frac{\mu_{t}}{0.85}(g_{i}\frac{\partial T}{\partial x_{j}}+g_{j}\frac{\partial T}{\partial xi})$$ (8)

Pressure strain term equation:

$$\Phi_{ij}=-0.18\rho \frac{\varepsilon }{k}(\bar{u_{i}^{\prime }u_{j}^{\prime }}-\frac{2}{3}k\delta_{ij})-0.6(P_{ij}-\frac{2}{3}P\delta_{ij})+f(k,\varepsilon ,n_{x},d)$$ (9)

Viscous dissipative term equation:

$$\varepsilon_{ij}=2\mu \bar{\frac{\partial u_{i}^{\prime }}{\partial x_{k}}\frac{\partial u_{j}^{\prime }}{\partial x_{k}}}$$ (10)

### 2.3 Numerical method and grid generation

In this study, the Finite Volume Method (FVM) is used to solve the problem, and the governing equations are discretized based on the pressure solver. The Mixture model was selected to study the distribution law of the flow field in the hydrocyclone and the Semi-Implicit-Method for Pressure-Linked Equations (SIMPLE) algorithm was used to solve the problem iteratively. The SIMPLE algorithm is a numerical method mainly used to solve incompressible fluids. Its core is to use the “guess-correction” process to calculate the pressure field on the basis of staggered grids, so as to solve the momentum equation [51]. Combined with the actual natural gas hydrate exploitation project, considering the influence of gravity on the cyclone separation process, the acceleration of gravity was set to 9.81m/s^2^. Set the total number of calculation steps to 10000 steps, and save 1 data file every 1000 steps. In order to ensure the accuracy of numerical simulation, the convergence accuracy is set to 10^−6^.

The fluid domain model of the in-situ sand removal hydrocyclone for natural gas hydrate solid fluidization exploitation was meshed, and five level grids were divided. In order to ensure the accuracy of calculation and reduce the amount of calculation, the grid independence test was carried out. Because of the complexity of fluid migration characteristics and mechanical distribution in the swirl field, in order to improve the stability and accuracy of numerical simulation and avoid false diffusion in the discretization process, local grid refinement was carried out around the separator pipe.

### 2.4 Boundary conditions

In this study, water was set as continuous phase and sand as discrete phase. The density of water is 998.2kg/m^3^, the viscosity is 0.001Pa·s, and the density of sand is 2700kg/m^3^. The boundary condition of the axial inlet of the hydrocyclone was set as the velocity inlet, and the incident velocity of the water phase and the sand phase is the same. The water outlet was set as outflow. The wall of the hydrocyclone was set as wall, with roughness of 0 and no-slip condition.

### 2.5 Simulation scenarios

In this study, the numerical simulation method was used to study the internal flow field characteristics and phase distribution law of hydrocyclone applicable to in-situ separation of sand phases in solid fluidized exploitation of natural gas hydrate. Based on the hydrocyclone model proposed by Chang et al [43]. and combined with the actual operating conditions of solid fluidization exploitation of natural gas hydrates, the effects of flow rate, flow ratio, sand volume fraction and sand particle diameter on the flow field characteristics in the hydrocyclone were studied. Numerical simulation scenarios are shown in Table 2. In 13 cases, Case1 was set as the basic case. Case1-4 study the distribution law of sand phase and water phase and tangential velocity field in swirling flow field under different flow conditions. Case5-7 and Case1 study the distribution of sand and water phases in the swirl field under different flow ratio. Case8-10 and Case1 study the effect of inlet sand content change on tangential velocity field and sand volume fraction distribution. Case 11–13 and Case 1 study the distribution of tangential velocity field and sand phase in swirling flow field under different sand particle diameters. In order to ensure the objectivity and accuracy of the research results, the parameter changes of each factor were uniformly distributed. At the same time, in order to avoid the interaction of various factors, the control variable method was used to study the effects of different factors on the flow field characteristics.

**Table 2:** Numerical simulation scenarios.

Scenario	Flow rate(m^3^/h)	Flow ratio(%)	Sand Volume fraction(%)	Sand particle diameter(μm)
Case1	4.83	5	20	80
Case2	3.83	5	20	80
Case3	2.83	5	20	80
Case4	1.83	5	20	80
Case5	4.83	10	20	80
Case6	4.83	15	20	80
Case7	4.83	20	20	80
Case8	4.83	5	10	80
Case9	4.83	5	30	80
Case10	4.83	5	40	80
Case11	4.83	5	20	20
Case12	4.83	5	20	40
Case13	4.83	5	20	60

**Fig 2:**
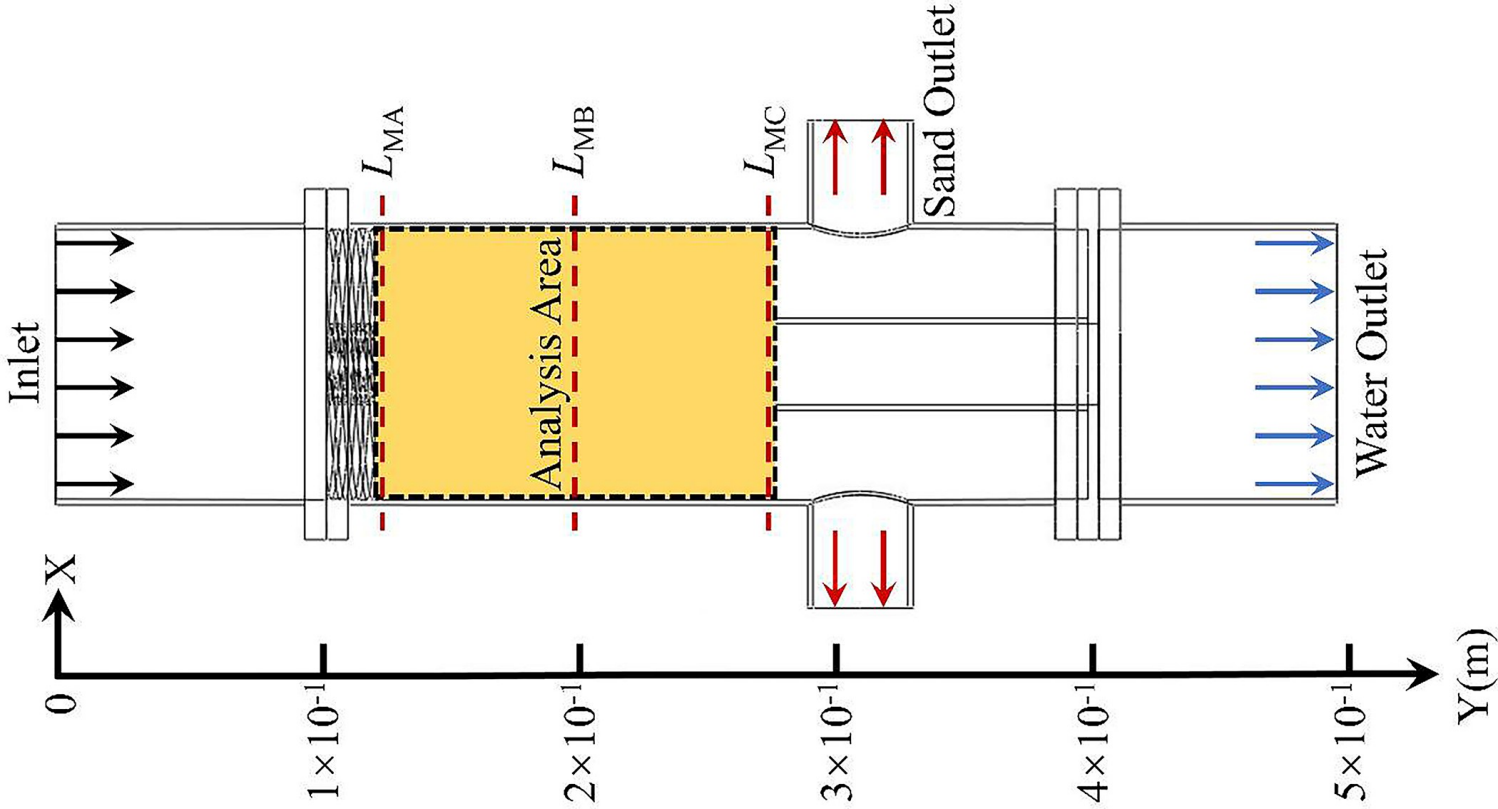
Analysis area and monitoring line position.

The water-sand two-phase mixture enters the device from the inlet of the hydrocyclone, and the mixture changes from axial motion to circular motion after passing through the spiral flow path. Due to the density difference between the water phase and the sand phase, the centrifugal force generated by the sand phase in the circular motion is large, so the sand migrates to the side wall of the hydrocyclone under the centrifugal force and is discharged from the device by the sand outlet, while the water phase is distributed in the axial center of the hydrocyclone and enters the water outlet through the separator pipe. Therefore, the flow field area between the spiral flow path and the separator pipe is the key area for water-sand separation. In order to more accurately study the variation characteristics and laws of the water-sand separation flow field area, three monitoring lines *L*_MA_, *L*_MB_, and *L*_MC_ are set equidistantly in this analysis area. According to the structural parameters of the hydrocyclone, the distances from the three monitoring lines to the inlet are 121mm, 200mm, and 279mm, respectively. The analysis area and the location of the monitoring line are shown in Fig 2.

In order to calculate the efficiency of sand discharge and water discharge in different cases. The dimensionless parameter *E* was defined as the efficiency evaluation value, which is expressed by the ratio of the mass flow rate at the outlet of each phase to the mass flow rate at the inlet. The calculation of sand discharge and water discharge efficiency of hydrocyclone are shown in Eqs (11)–(12).


$$E_{s}=\frac{m_{so}}{m_{si}}$$ (11)

$$E_{w}=\frac{m_{wi}}{m_{wo}}$$ (12)

Finally, by analyzing the variation trend of velocity field, sand phase volume fraction and water phase volume fraction on the analysis area and monitoring lines, the influence of various factors on the internal flow field characteristics and distribution law of each phase of the hydrocyclone were obtained. In this study, in order to ensure the accuracy of the visibility of numerical simulation results, Ansys post-processing software was used to process the simulation results, and the ratio of icon size to model size is 1:1.

## 3. Result and discussion

### 3.1 Grid independence

**Fig 3:**
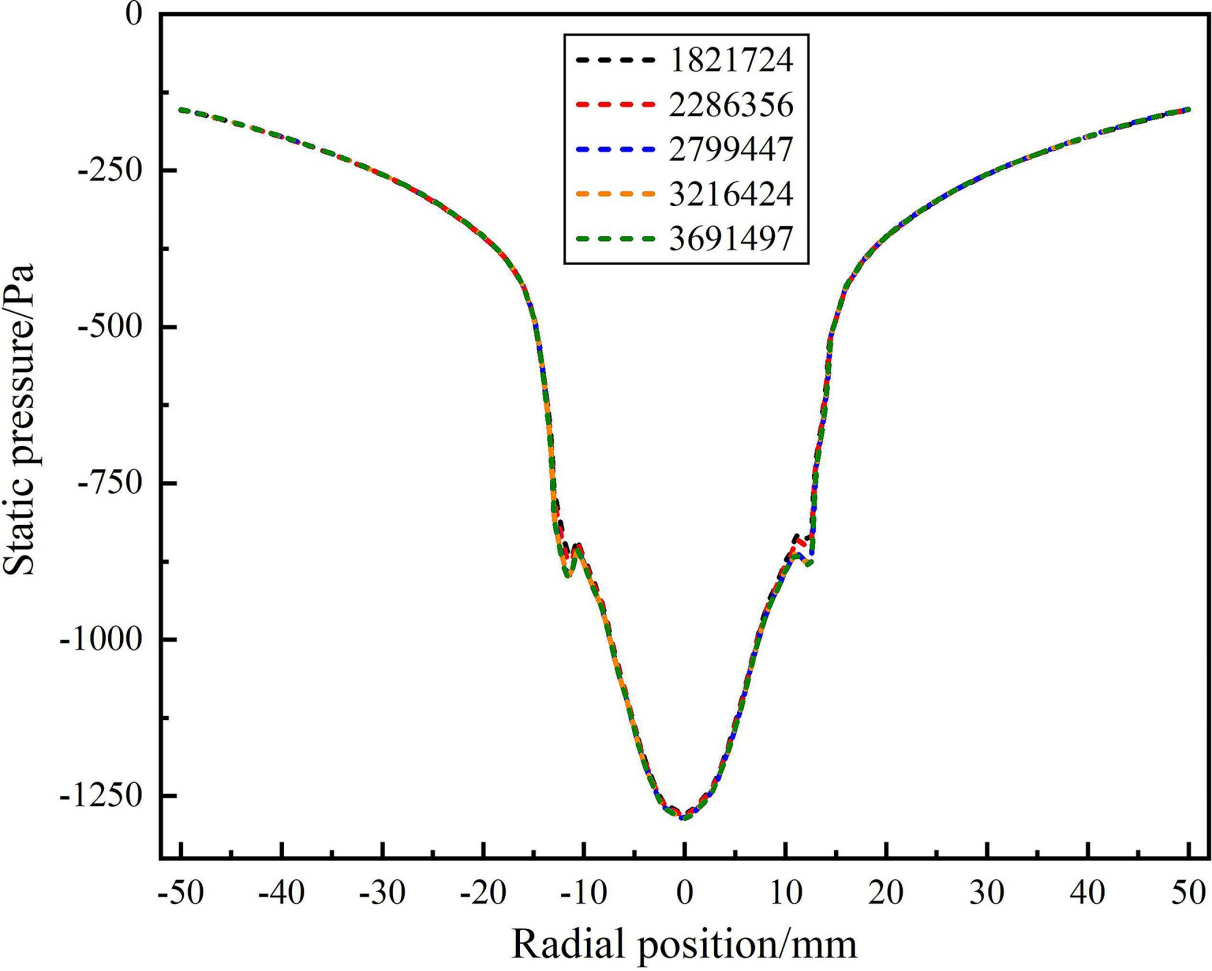
Grid independence verification.

**Fig 4:**
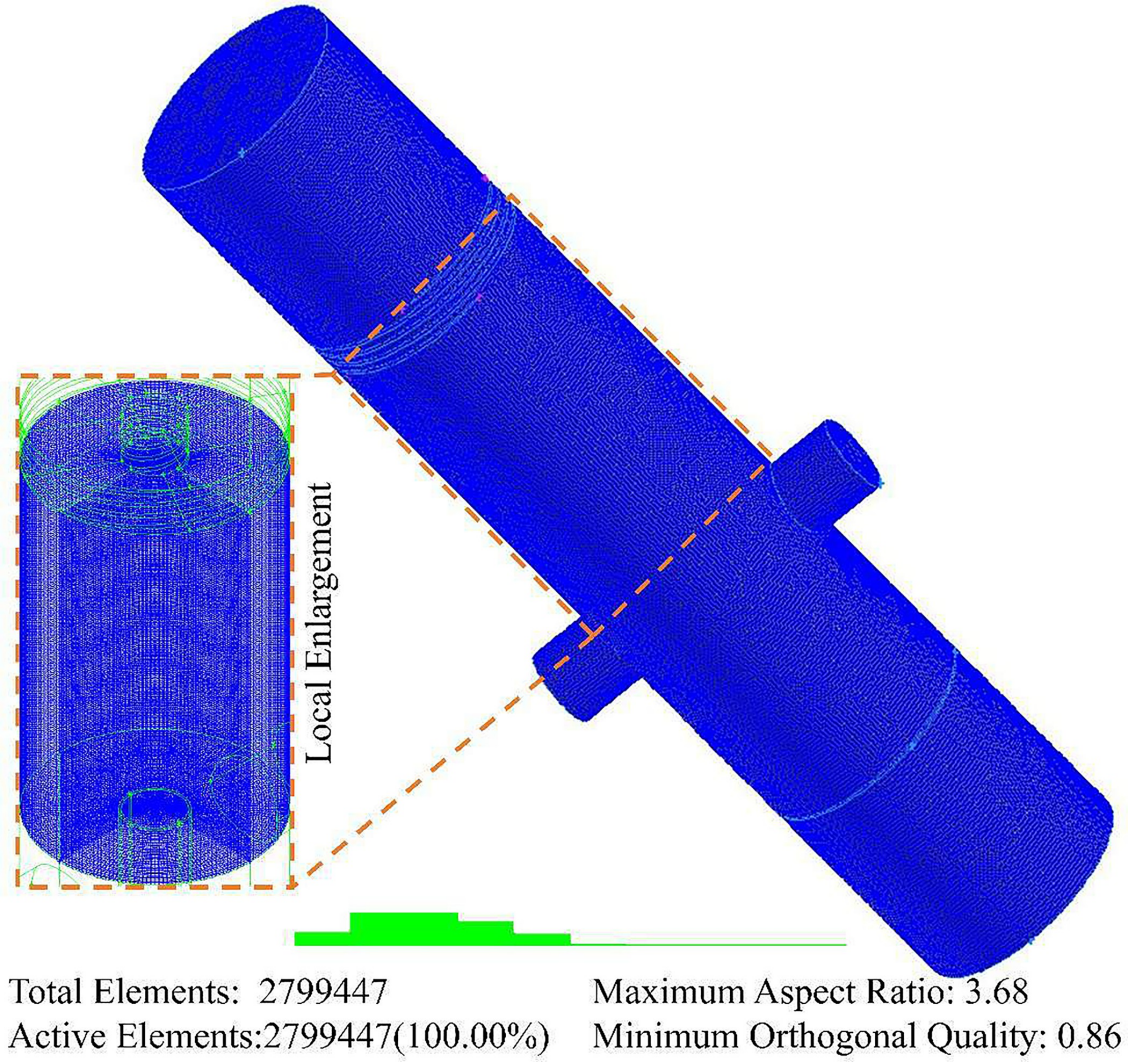
Schematic diagram of grid generation.

The Ansys’s pre-processing software Gambit was used to grid the fluid domain model of hydrocyclone which is applicable to sand removal in solid fluidization exploitation of natural gas hydrate, five levels of grids with 1821724, 2286356, 2799447, 3216424 and 3691497 cells were examined. The static pressure distribution on the monitoring line *L*_MC_ under different grid levels was analyzed, as shown in Fig 3. When the grid number reached 2799447, the static pressure on the monitoring line *L*_MC_ is basically unchanged [51]. In order to ensure the accuracy of numerical simulation and save calculation time, the fluid domain model of hydrocyclone is divided into 2799447 grid elements, and the meshing results is shown in Fig 4.

### 3.2 Model validation

**Fig 5:**
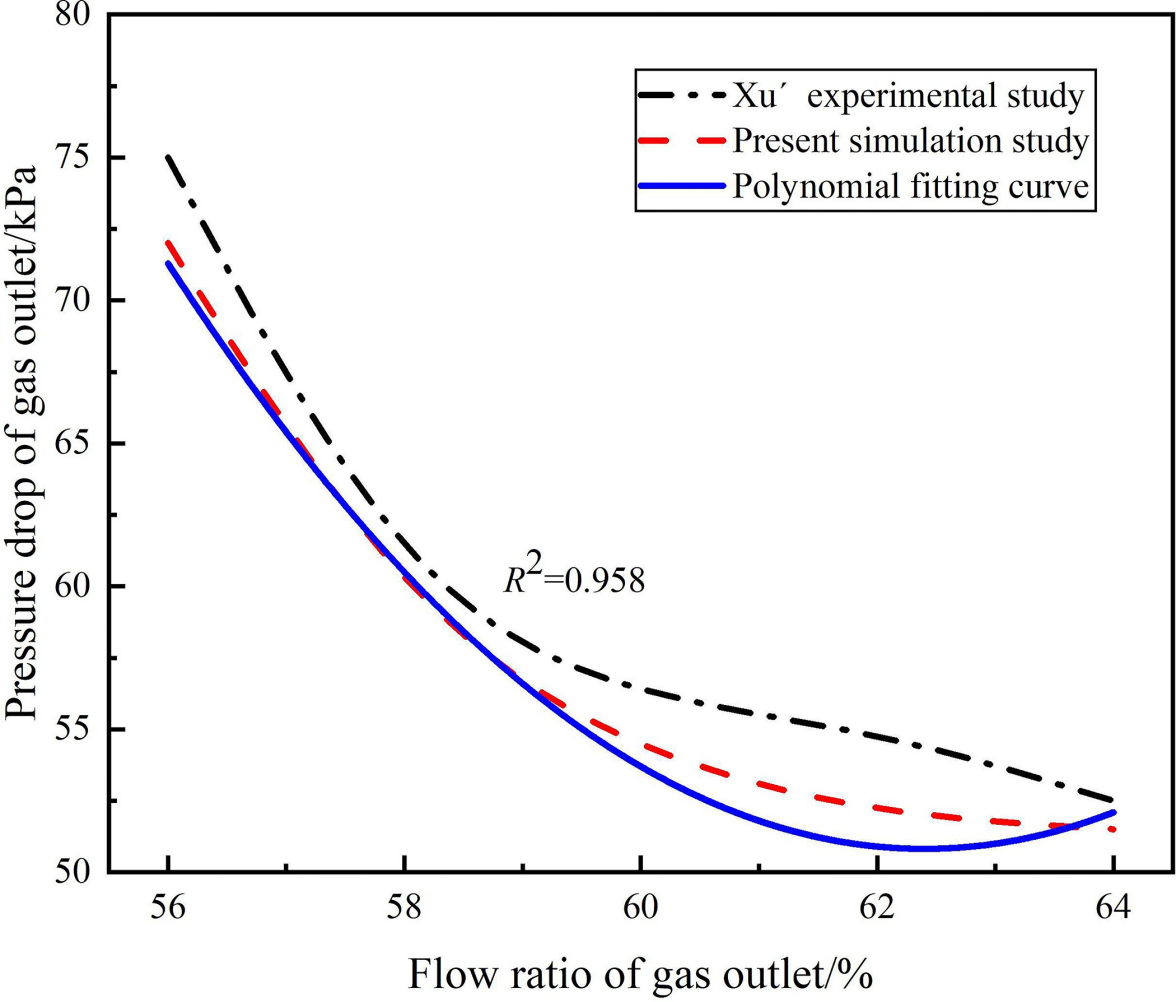
Gas outlet pressure drop under different flow ratios.

**Fig 6:**
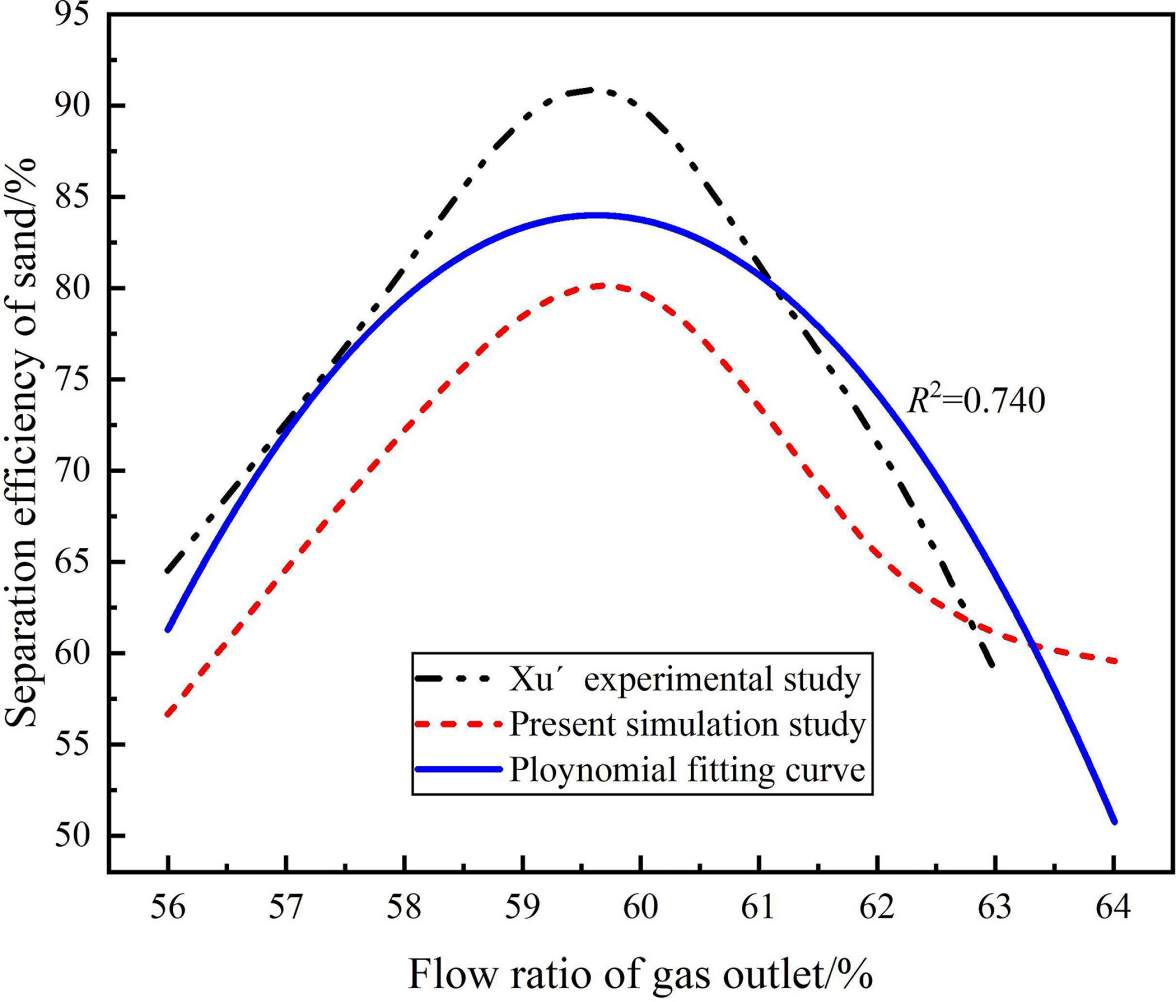
Sand phase separation efficiency under different flow ratios.

In this study, Xu’s experiments were used to verify the accuracy of numerical simulation and turbulence model [52]. In Xu’s experimental research, the experimental platform was designed according to the actual working conditions, so this paper re-establish the numerical simulation calculation model based on Xu’s experiment. The structural parameters of the hydrocyclone are shown in Tables 1 and 3. In the experiment, the distribution law of pressure drop and sand phase separation efficiency under the condition of inlet flow rate of 4.83m^3^/h and gas outlet flow ratio of 56%-64% were studied. Therefore, the same physical model and boundary conditions as the experimental device were establish, and the Mixture model and Reynolds stress model were used to carry out numerical simulation. The gas outlet pressure drop distribution and sand phase separation efficiency obtained from numerical simulation and experiment under the condition of gas outlet flow ratio of 56%-64% are shown in Figs 5 and 6. It can be seen from Figs 5 and 6 that due to the simplification of the real process in the numerical simulation process, the numerical simulation results are generally smaller than the experimental results. By polynomial fitting between the numerical simulation results and the experimental results, the fitting degree *R*_2_ of the gas phase outlet pressure drop between the experimental data and the numerical simulation data is 0.958, and the fitting degree *R*^2^ of the sand phase separation efficiency between experimental data and the numerical simulation data is 0.740, which prove the accuracy of the numerical simulation and the turbulence model.

**Table 3:** Experimental device structure parameters.

Structure	Swirl chamber length (m)	Main diameter (m)	Inverted cone height (m)	Drain hole height (m)	Inlet area (m^2^)
Parameter	2.38×10^−1^	4.5×10^−2^	9.6×10^−2^	0.1×10^−1^	1.4×10^−3^*1.4×10^−2^

### 3.3 Flow field characteristics and phase distribution laws under different parameters

In order to study the effects of different parameters on the flow field characteristics and phase distribution law and the separation efficiency of hydrocyclone, the numerical simulation method was used to study the influence of different flow rate, different flow ratio, different sand volume fraction and different sand particle diameter on the velocity field distribution and the sand phase and water phase distribution law.

#### 3.3.1 Flow field characteristics and distribution rules under different flow rate

**Fig 7:**
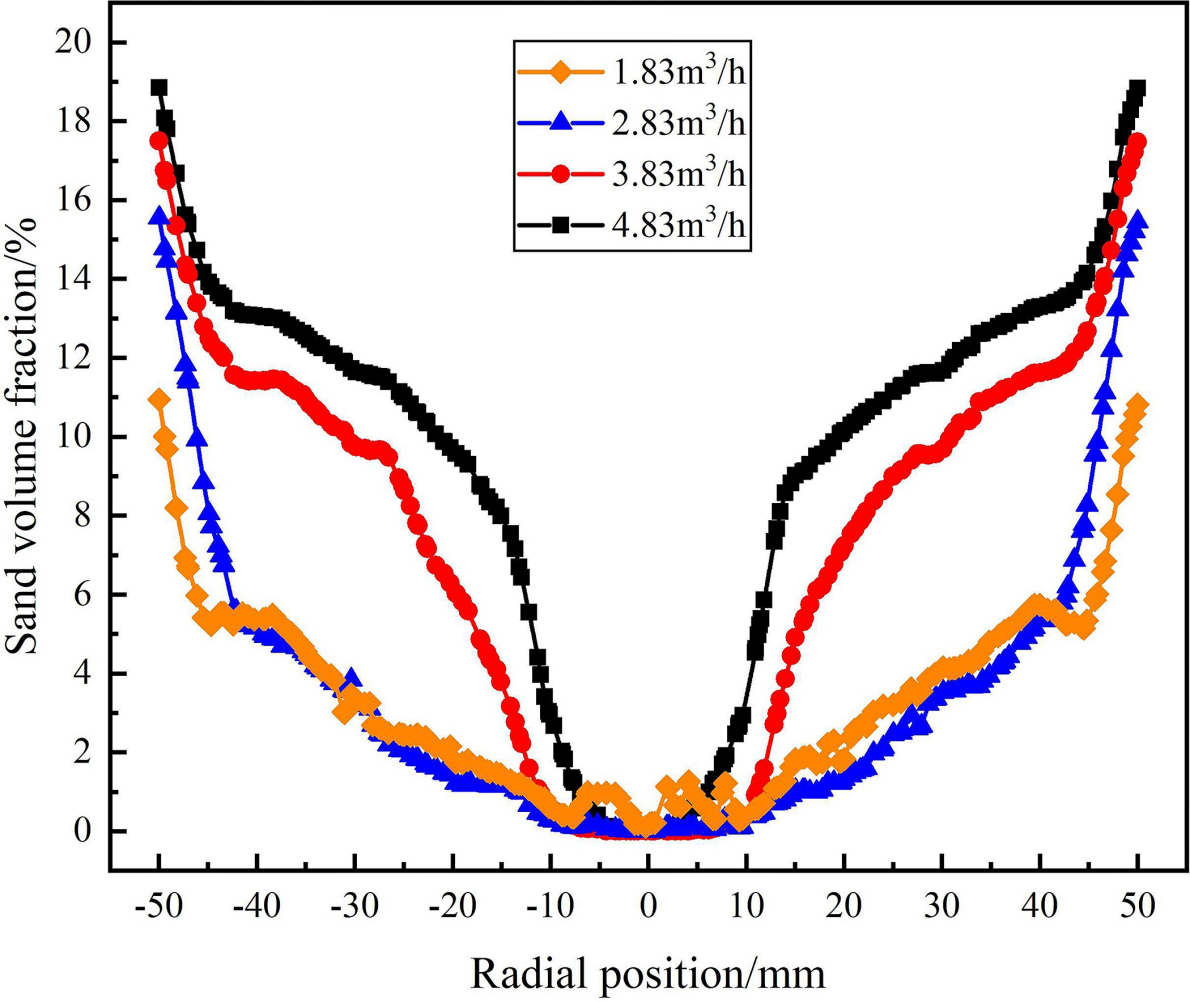
Distribution of sand phase on monitoring line *L*_MA_ under different flow rate conditions.

**Fig 8:**
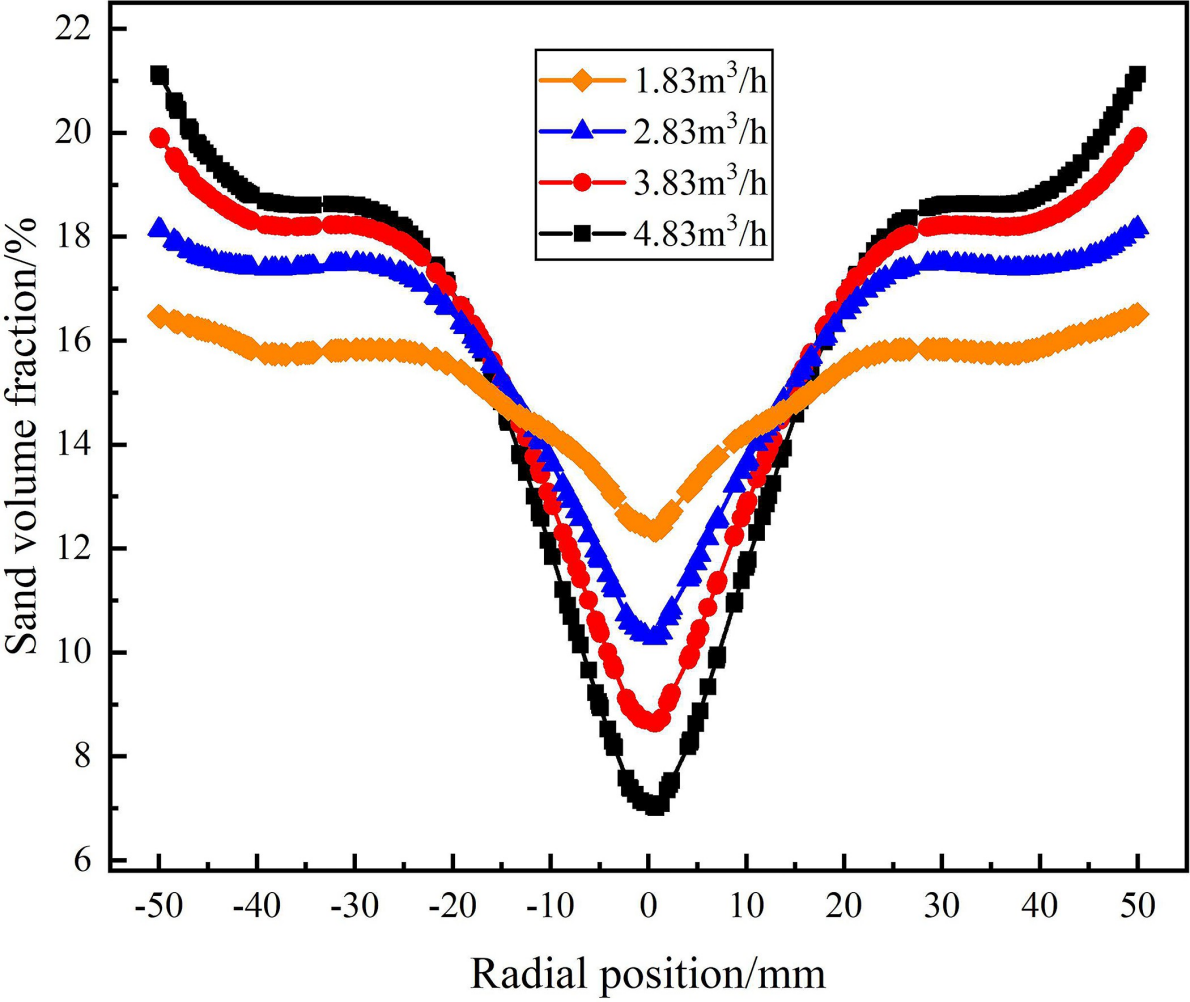
Distribution of sand phase on monitoring line *L*_MB_ under different flow rate conditions.

**Fig 9:**
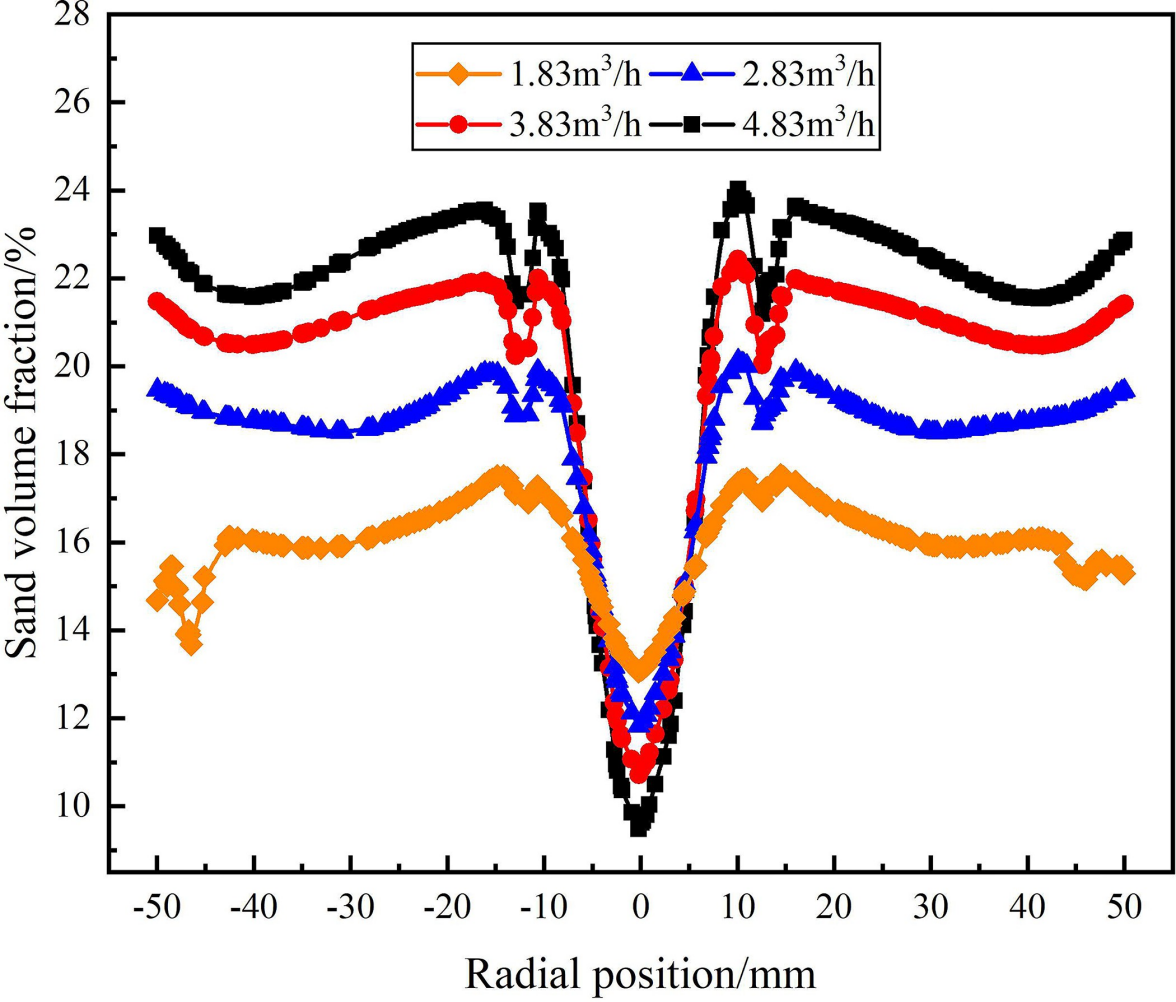
Distribution of sand phase on monitoring line *L*_MC_ under different flow rate conditions.

The distribution of sand phase on monitoring line *L*_MA_, *L*_MB_, and *L*_MC_ under different flow conditions are shown in Figs 7–9. It can be seen from Fig 7 that on the monitoring line *L*_MA_, the volume fraction of sand phase gradually increases in the radial position range of 0mm-±50mm. When the flow rate is 4.83m^3^/h, the sand fraction reaches its maximum, the sand volume fraction reaches a minimum of 10.5% at the radial position ±50mm. As the flow rate increased from 1.83m^3^/h to 4.83m^3^/h, the sand volume fraction at the inner wall of the hydrocyclone increased from 10.5% to 19%. It is proved that as the flow rate increases, the tangential velocity in the flow field increases, and the sand phase migrates to the flow field edge more significantly in the process of separation.

The distribution of sand volume fraction on monitoring line *L*_MB_ under different flow conditions is shown in Fig 8. It can be seen from Fig 8 that on monitoring line *L*_MB_, the sand volume fraction at the inner wall of the hydrocyclone still increases with the increase of flow rate, but the sand volume fraction at the axis decreases with the increase of flow rate. As the axial distance increases from 121 mm to 200 mm from the inlet, the sand volume fraction at the radial position ±50 mm in the flow field gradually increases. When the flow rate is 4.83m^3^/h, the sand volume fraction reaches a maximum of 21.2% at the edge of the flow field, and the sand volume fraction reaches a minimum of 6.7% at the axial center of the flow field. When the flow rate is 1.83m^3^/h, the sand volume fraction at the edge of the flow field is 16.3%, and the sand volume fraction at the axis of the flow field is 12.4%.

The distribution of sand volume fraction on monitoring line *L*_MC_ is shown in Fig 9. From Fig 9, it can be seen that under the flow rate conditions of 1.83m^3^/h-4.83m^3^/h, the sand volume fraction rapidly decreases at the axial position of 279mm and the radial position of ±10mm, and all reach their minimum at the radial position of 0mm. The flow rate increased from 1.83m^3^/h to 4.83m^3^/h, and the sand volume fraction at the axis center of the flow field decreased from 13% to 9.2%. When the flow rate is in the range of 2.83m^3^/h-4.83m^3^/h, the sand volume fraction still shows an increasing trend at the radial position ±50mm. Compared with the monitoring line *L*_MB_, the sand volume fraction at the edge of flow field increases by 1.8%, 1.8% and 1.5% respectively under the conditions of 2.83m^3^/h, 3.83m^3^/h and 4.83m^3^/h. However, when the flow rate is 1.83m^3^/h, the sand volume fraction at the edge of flow field decreases by 0.4%. It is proved that when the flow rate is in the range of 2.83m^3^/h-4.83m^3^/h, the tangential velocity in the flow field increases gradually from the axial position 121mm to 279mm. When the flow rate is 1.83m^3^/h, the tangential velocity decreases gradually within the 200mm-279mm range of the axial position of the flow field, and the separation effect on the sand phase is gradually decreased.

**Fig 10:**
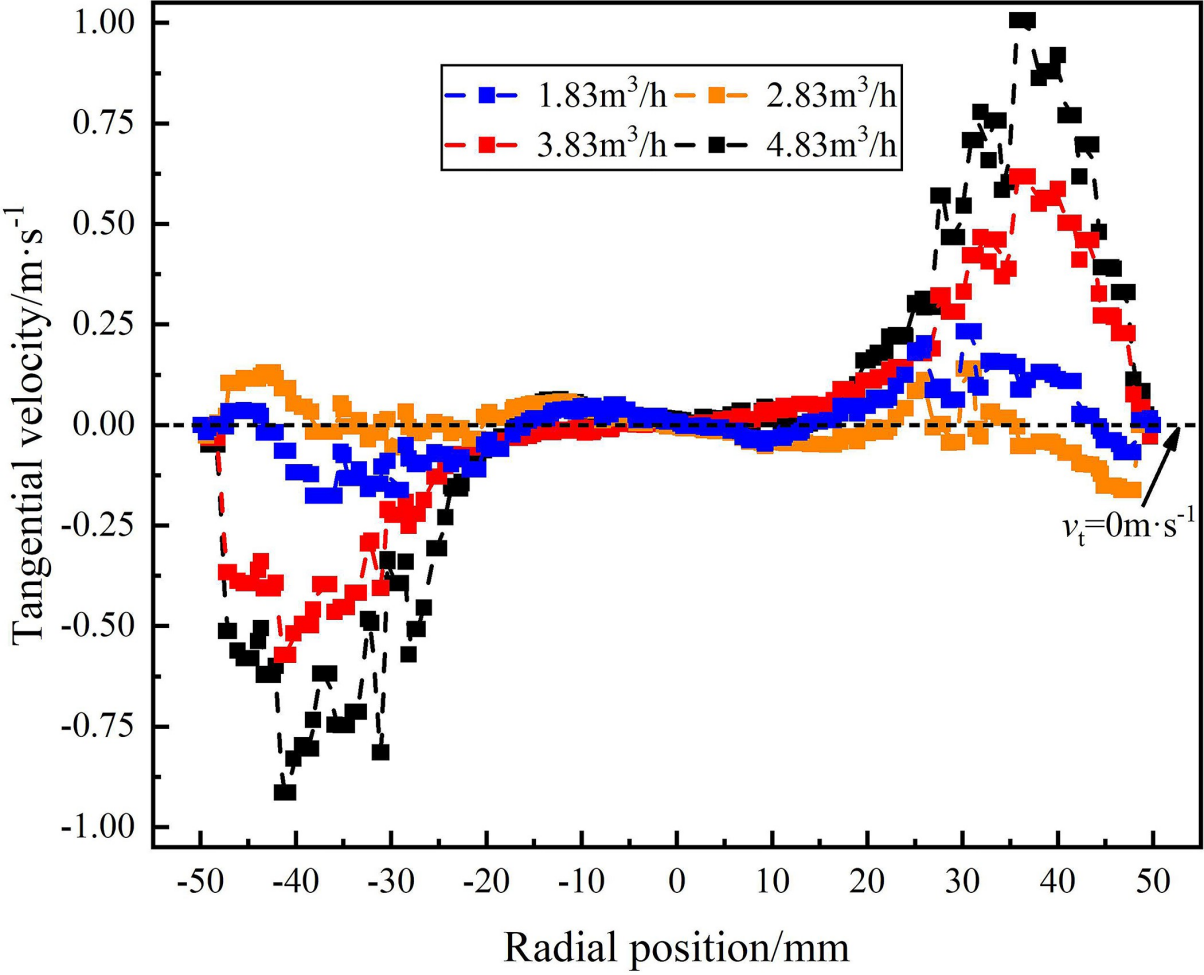
Distribution of tangential velocity on monitoring line *L*_MA_ under different flow rate conditions.

**Fig 11:**
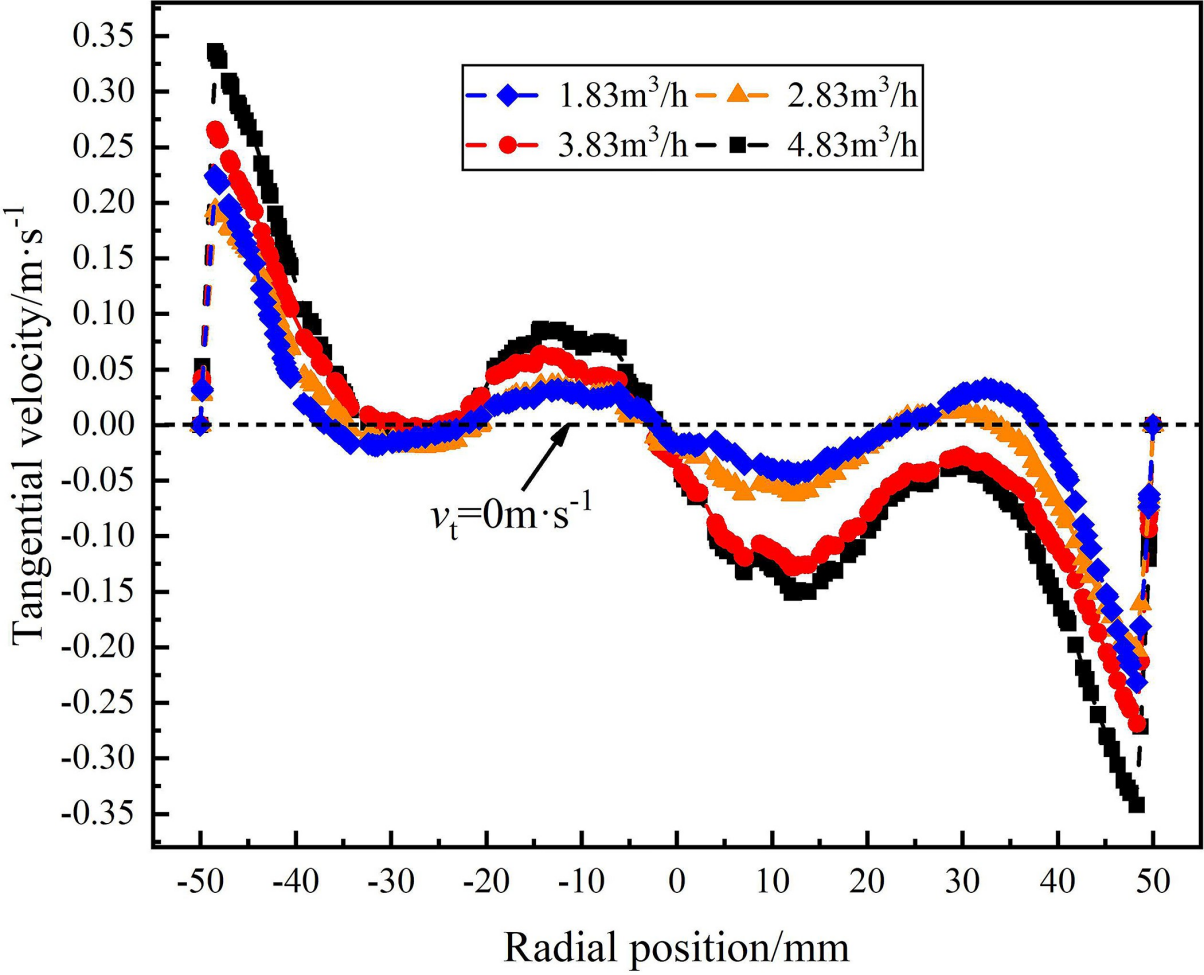
Distribution of tangential velocity on monitoring line *L*_MB_ under different flow rate conditions.

**Fig 12:**
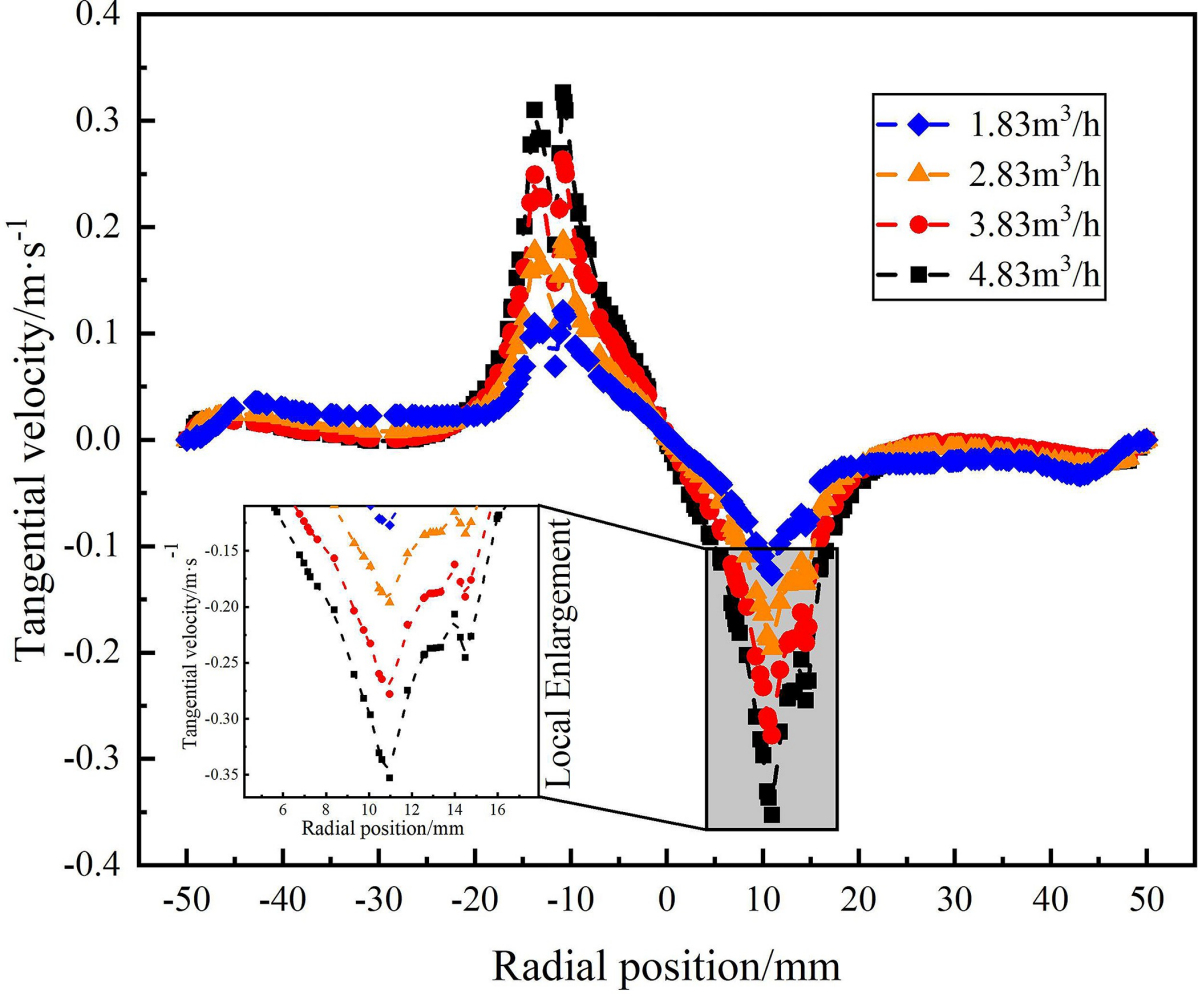
Distribution of tangential velocity on monitoring line *L*_MC_ under different flow rate conditions.

The tangential velocity distribution on monitoring line under different flow conditions are shown in Figs 10–12. Fig 10 shows the tangential velocity distribution on monitoring line *L*_MA_. It can be seen from Fig 10 that the tangential velocity on monitoring line *L*_MA_ shows a symmetrical distribution characteristic and takes the radial position 0 mm as the axis of symmetry that first increases and then decreases from the radial position 0 mm to ±50 mm. The reason why the tangential velocity decreases at the side wall is that the wall roughness was set to 0 and there was no slip during the numerical simulation. The maximum tangential velocity under the four flow conditions is greater than 0m/s, indicating that part of the axial velocity of the mixture changes into a tangential velocity after passing through the spiral flow path. When the flow rate is 4.83m^3^/h, the maximum tangential velocity is 1m/s, which appears at the radial position of ±40mm. When the flow rate is 1.83m^3^/h, the maximum tangential velocity of 0.23m/s appears at the radial position of ±30mm. It shows that with the increase of the flow rate, the axial velocity at the inlet increases, which leads to the increase of the tangential velocity of the fluid after passing through the spiral flow path.

Fig 11 shows the tangential velocity distribution on monitoring line *L*_MB_ under the flow condition of 1.83m^3^/h-4.83m^3^/h. As the axial distance from the inlet increases from 121mm to 200mm, the maximum tangential velocity at the edge of flow field decreases under different flow conditions, but the tangential velocity gradually increases within the range of -20mm-20mm in the radial position. When the flow rate is 4.83m^3^/h, the tangential velocity reaches a maximum of 0.33m/s at the edge of flow field. Compared with the monitoring line *L*_MA_, the maximum tangential velocity decreases 0.67m/s. Similarly, when the traffic is 1.83m^3^/h and 3.83m^3^/h, the maximum tangential velocity decreases 0.35m/s and 0.01m/s, respectively. It is proved that as the axial distance increases, the tangential velocity in the swirling flow field decreases gradually, and the separation effect of the swirling flow gradually weakens.

Fig 12 shows the tangential velocity distribution on monitoring line *L*_MC_ under different flow conditions. It can be seen from Fig 12 that the maximum value of the tangential velocity in the flow field under different flow conditions at the axial position of 279mm appears at the radial position of 10.8 mm. According to the local enlargement, the maximum tangential velocity of 1.83m^3^/h, 2.83m^3^/h, 3.83m^3^/h and 4.83m^3^/h at the radial position 108mm is 0.13m/s, 0.19m/s, 0.27m/s and 0.35m/s, respectively. The maximum tangential velocity still shows the distribution law that increase with the increase of flow rate. However, compared with the monitoring line *L*_MA_, the tangential velocity decreases by 0.65m/s, 0.34m/s and 0.1m/s respectively under the flow conditions of 1.83m^3^/h, 3.83m^3^/h and 4.83m^3^/h. When the flow rate is 2.83m^3^/h, the maximum tangential velocity remains unchanged. It shows that with the increase of the axial distance, the tangential flow produced by the spiral flow path gradually weakens, and the intensity of the swirl flow in the flow field gradually weakens.

**Fig 13:**
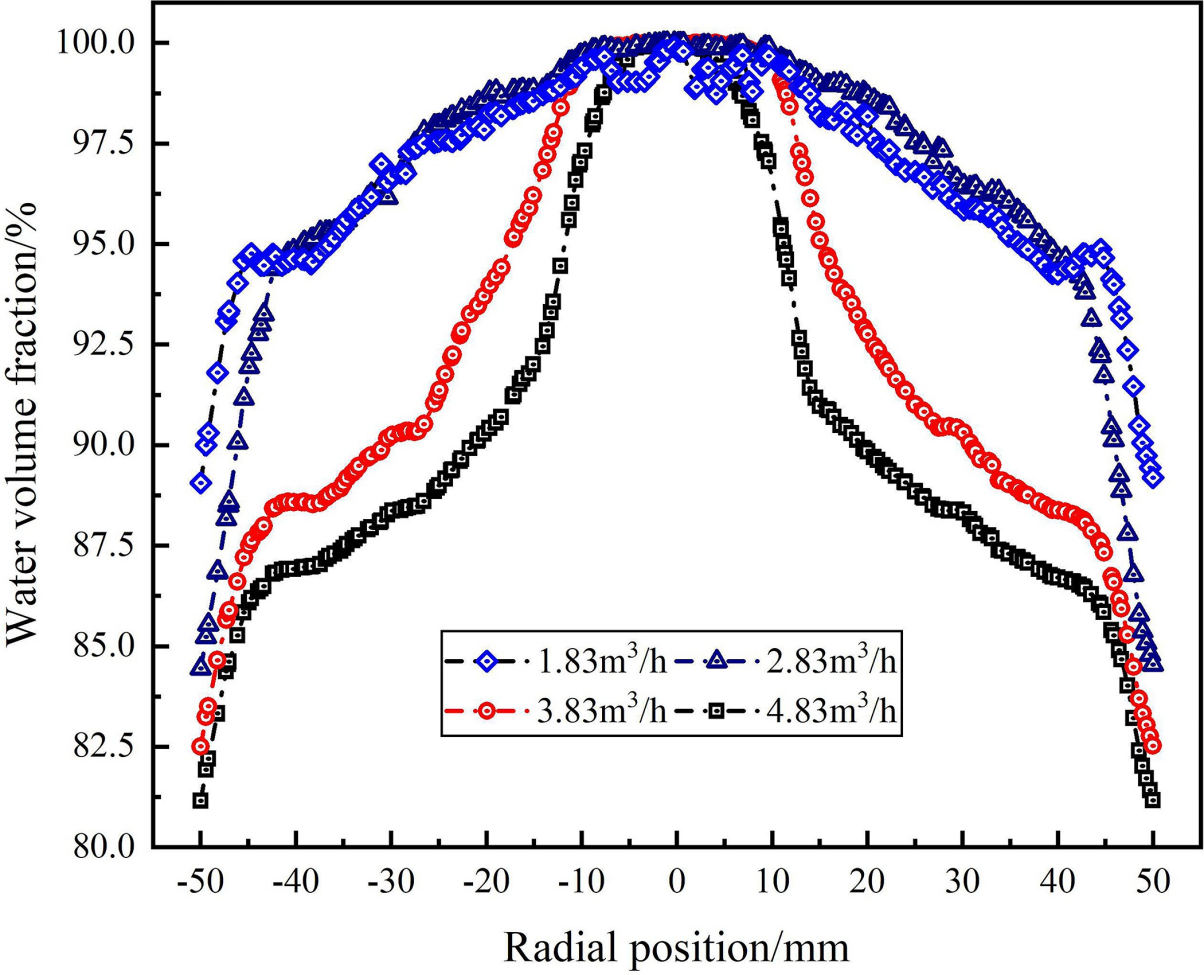
Distribution of water phase on monitoring line *L*_MA_ under different flow rate conditions.

**Fig 14:**
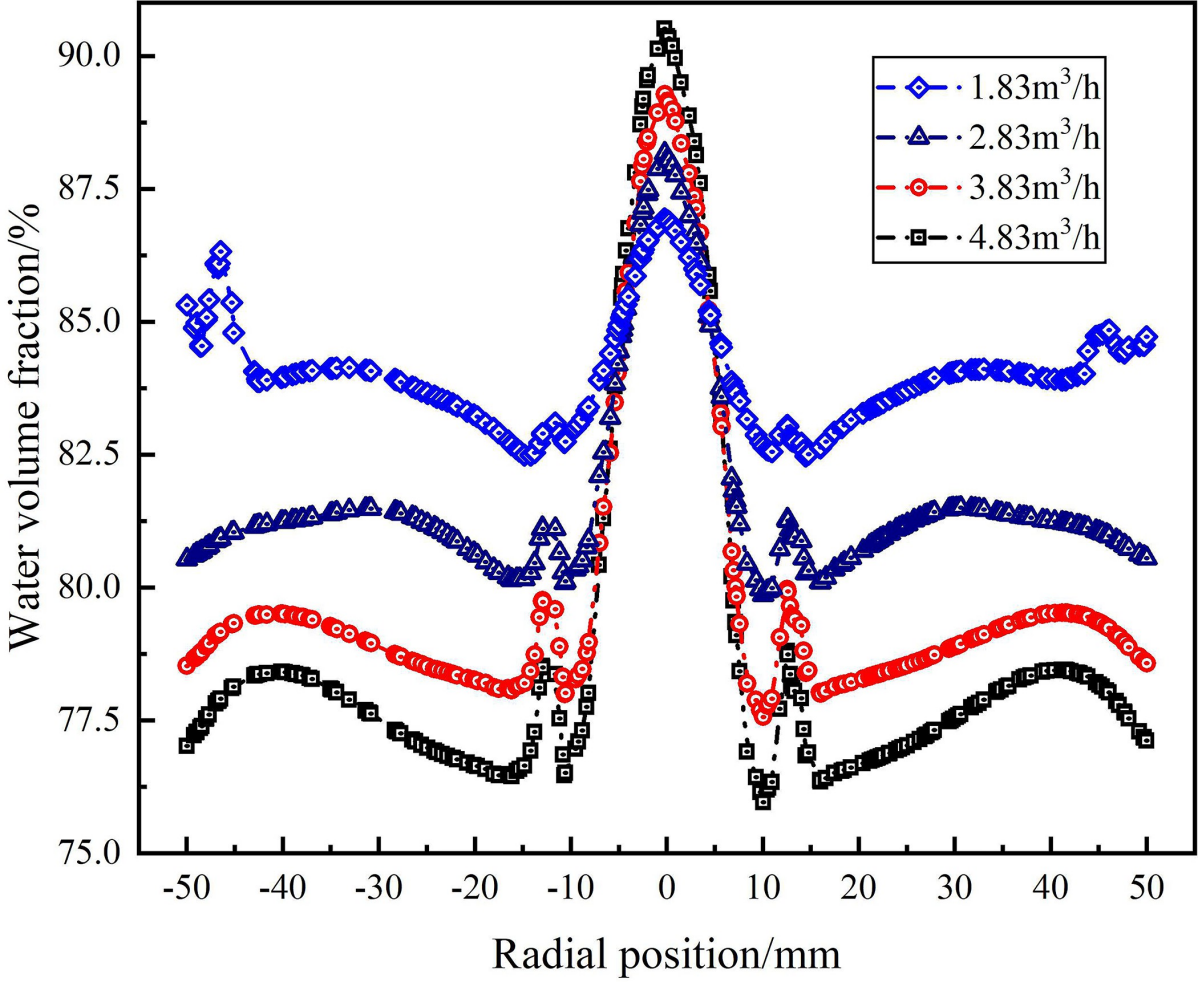
Distribution of water phase on monitoring line *L*_MB_ under different flow rate conditions.

The water phase distribution in the swirling flow field under different flow conditions are shown in Figs 13, 14. As shown in Fig 13, when the flow rate is in the range of 1.83m^3^/h-4.83m^3^/h, the water volume fraction on monitoring line *L*_MA_ reaches 100% at the radial position of 0mm and the water volume fraction decreases gradually from the radial position of 0mm to ±50 mm under each flow rate condition. Among them, when the flow rate is 3.83m^3^/h and 4.83m^3^/h, the water volume fraction is concentrated in the range of radial position ±24mm, and the concentration of water phase is more significant with the increase of flow rate. At the radial position ±50mm, the water volume fraction decreases with the increase of flow rate. When the flow rate increases from 1.83m^3^/h to 4.83m^3^/h, the water volume fraction at the edge of flow field decreases from 88.8% to 81.2%. It shows that the tangential velocity in the flow field increases with the increase of the flow rate. Since the density of the water phase is smaller than that of the sand phase, according to the principle of cyclone separation, the distribution of the water phase is closer to the center of the flow field.

The water volume fraction distribution on monitoring line *L*_MC_ under different flow rate conditions is shown in Fig 14. As can be seen from Fig 14, compared with the monitoring line *L*_MA_, the water phase distribution on monitoring line *L*_MC_ is more concentrated to the center of the flow field, and the concentrated distribution range of the water phase does not change significantly under different flow conditions, all in the range of radial position ±10mm. The water volume fraction at the radial position 0mm increases with the increase of the flow rate, the flow rate increases from 1.83m^3^/h to 4.83m^3^/h, and the water volume fraction at the center of the flow field is 86.9%, 88.1%, 89.2% and 90.3%, respectively. The water volume fraction at the radial position ±50mm decreases with the increase of flow rate, the flow rate increases from 1.83m^3^/h to 4.83m^3^/h, and the water volume at the edge of flow field is 85.3%, 80.5%, 78.5% and 77%, respectively. It shows that at the axial distance of 279 mm from the inlet, the volume fraction of the water is significantly changed by the flow rate, but the distribution area of the water phase is not affected by the flow rate.

#### 3.3.2 Flow field characteristics and distribution rules under different flow ratio

**Fig 15:**
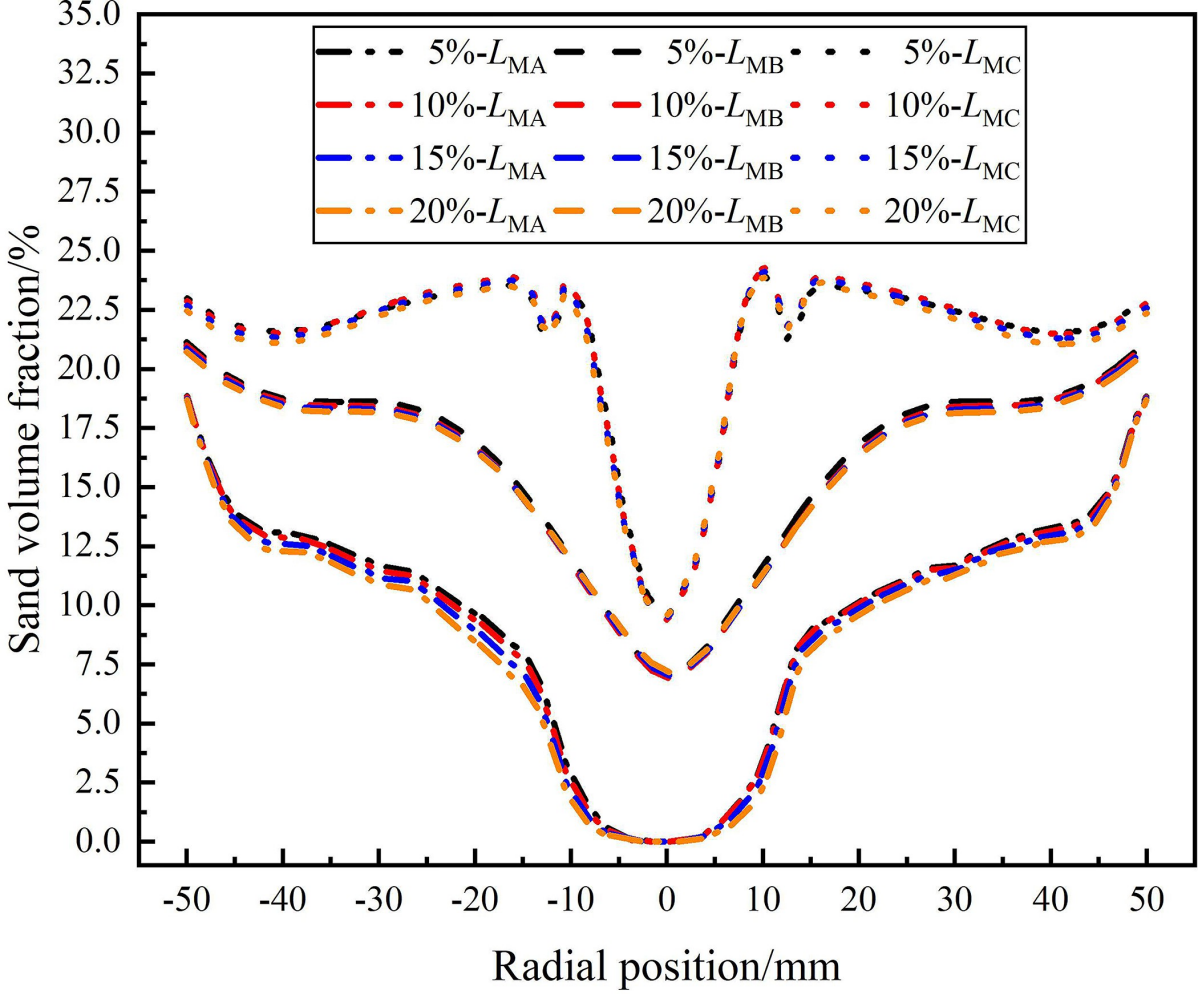
Distribution of sand phase on monitoring lines under different flow ratio conditions.

The distribution of sand volume fraction on monitoring lines *L*_MA_, *L*_MB_, and *L*_MC_ under different flow ratio conditions is shown in Fig 15. It can be seen from Fig 15 that at different monitoring lines, the sand volume fraction shows a gradually increasing distribution law in the radial position range of 0 mm to ±50 mm. On monitoring line *L*_MA_, the sand volume fraction reaches a minimum of 0% at the axial center of the flow field and reaches a maximum of 18.8% at the edge of the flow field. On monitoring line *L*_MC_, the sand volume fraction reaches a minimum of 8.9% at the axial center of the flow field and reaches a maximum of 22.6% at the edge of the flow field. That is, within the axial range of 121mm-279mm from the inlet, the sand volume fraction at the axial center and edge of the flow field increases by 8.9% and 3.8%, respectively. However, on the same monitoring line, the flow ratio of the sand outlet increases from 5% to 20%, and there is no significant difference in the sand volume fraction. It shows that the flow ratio of the sand outlet has no significant effect on the separation of the sand phase.

**Fig 16:**
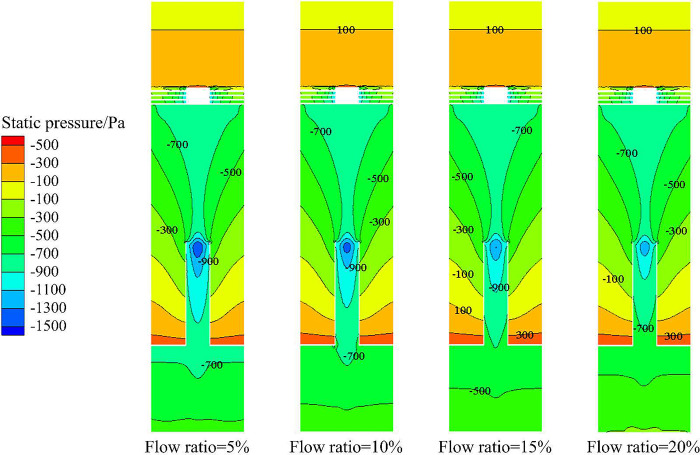
Static pressure distribution in X-Y section under different flow ratio conditions.

The static pressure distribution in X-Y section under different flow ratios is shown in Fig 16. It can be seen from Fig 16 that under different flow ratio conditions, the static pressure in X-Y section is roughly the same and the distribution shows a symmetrical distribution law with the radial position 0mm as the symmetrical axis. As the radial position increases from 0mm to 50mm, the static pressure gradually increases, resulting in a gradual increase in the sand volume fraction from the axis to the side wall. At the same time, the static pressure increases with the increase of axial distance, which leads to the increase of sand volume fraction from the monitoring line *L*_MA_ to the monitoring line *L*_MC_.

**Fig 17:**
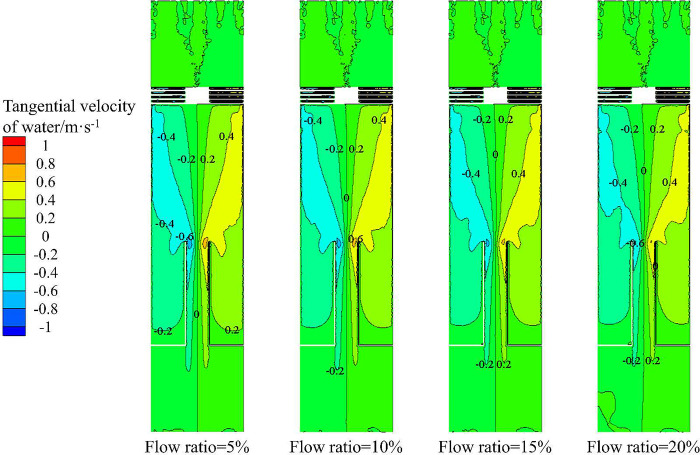
Tangential velocity distribution of water in X-Y section under different flow ratio conditions.

**Fig 18:**
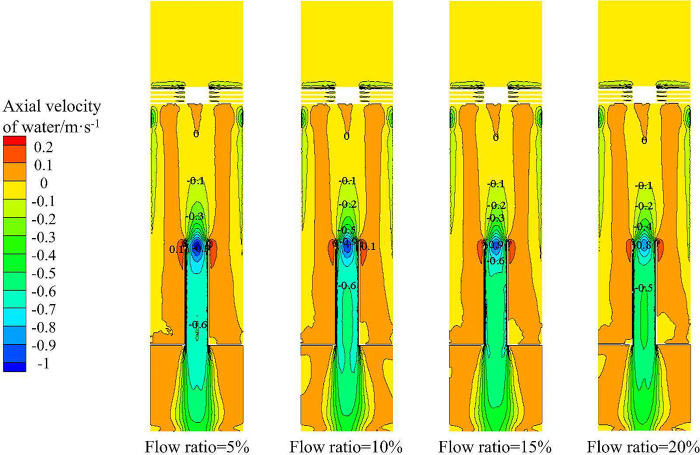
Axial velocity distribution of water in X-Y section under different flow ratio conditions.

Figs 17 and 18 show the tangential velocity distribution and axial velocity distribution of the water phase in the X-Y section under different flow ratios, respectively. From Fig 17, it can be seen that the tangential velocity distribution in the X- Y section increases gradually from the center to the side wall. Therefore, the distribution of sand volume fraction in the flow field increases gradually from the center to the side wall under the influence of tangential velocity. With the increase of axial distance, the distribution area with tangential velocity of 0–0.2 m·s^-1^ decreases, and the distribution area with tangential velocity of 0.4–0.6 m·s^-1^ moves to the axis. Combined with Fig 15, it can be seen that the area where the volume fraction of sand decreases gradually decreases in the radial direction.

It can be seen from Fig 18 that the axial velocity at the axial position in the X-Y section is negative, indicating that the water phase is distributed at the axis and migrates upward. The axial velocity on the outside of the separation tube is positive, which proves that the sand phase are mainly distributed here and migrate downward. However, there is no significant difference in the axial velocity distribution at the same monitoring line under the condition of different flow ratio, which is the same as the sand phase distribution shown in Fig 15.

#### 3.3.3 Flow field characteristics and distribution rules under different sand content

**Fig 19:**
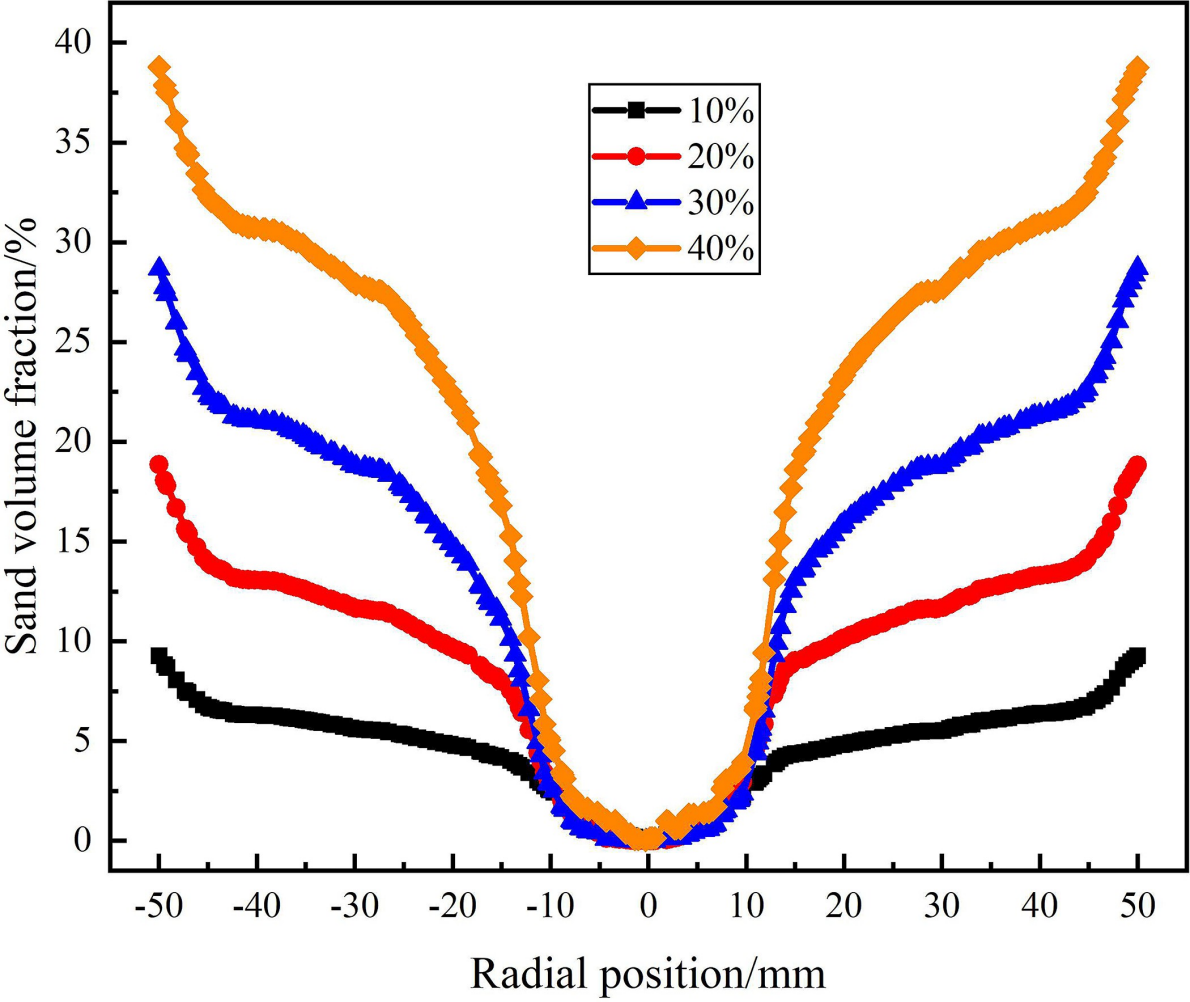
Distribution of sand phase on monitoring line *L*_MA_ under different sand content conditions.

**Fig 20:**
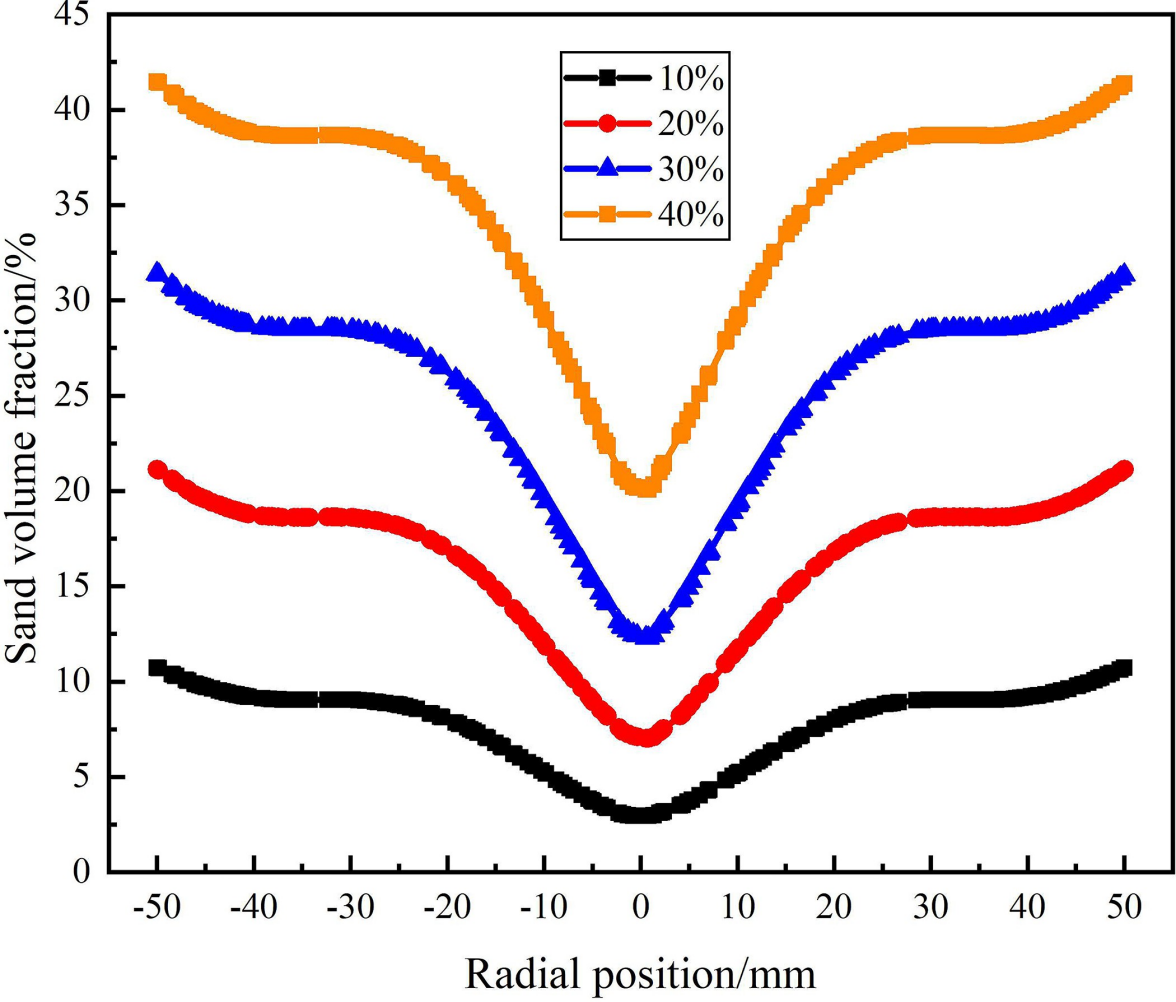
Distribution of sand phase on monitoring line *L*_MB_ under different sand content conditions.

**Fig 21:**
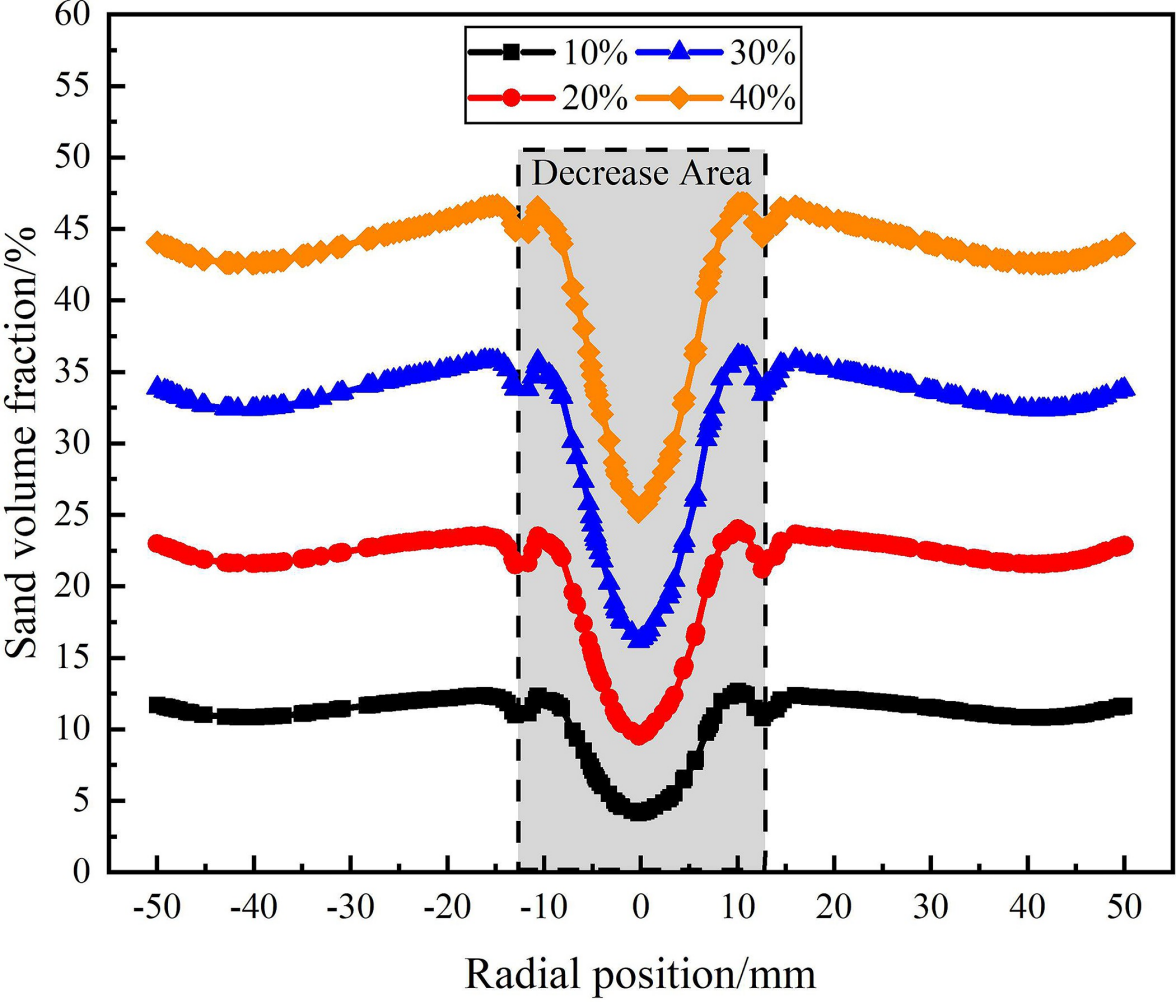
Distribution of sand phase on monitoring line *L*_MC_ under different sand content conditions.

The distribution of sand phase in the analysis area under different sand volume fraction is shown in Figs 19–21. Fig 19 shows the s distribution of and volume fraction at the on monitoring line *L*_MA_. As can be seen from Fig 19, at the axial distance 121mm from the inlet, the sand content is in the range of 10% to 40%, and the sand distribution in the flow field shows a symmetrical distribution characteristic with the radial position 0mm as the symmetry axis. In the range of 0mm-±50mm in the radial position, the sand volume fraction increases gradually, indicating that the sand volume fraction gradually increases from the center to the edge of the flow field. This is because the centrifugal force in the circular motion increases with the increase of the radial distance, which leads to the migration of the sand phase to the side wall. The sand volume fraction at the center of the flow field is 0%, and the sand volume fraction at the edge of the flow field increases with the increase of sand content. This is due to the fact that in the process of cyclone separation, with the increase of sand content, the more sand phase migrates to the side wall under the action of centrifugal force. When the sand content is 40%, the sand volume fraction at the edge of the flow field reaches a maximum of 38.8%, which is 29.5%, 20%, 10.1%higher than that under the conditions of sand content of 10%, 20%, 30%.

The distribution of sand volume fraction on monitoring line *L*_MB_ under different sand content conditions is shown in Fig 20. It can be seen from Fig 20 that on monitoring line *L*_MB_, the sand volume fraction in the swirling flow field under different sand content conditions still shows a distribution law that gradually increases from the center to the edge of flow field. However, there are significant differences in the sand volume fraction under different sand contents, and the sand volume fraction in the flow field increases with the increase of sand content. As the sand content increases from 10% to 40%, the sand volume fraction at the center of the swirling field increases from 2.9% to 20.1%, and the sand volume fraction at the edge of the swirling field increases from 10.7% to 41.5%. This is because as the axial distance increases, the tangential velocity in the flow field gradually decreases, leading to a gradual weakening of centrifugal force, which in turn leads to incomplete migration of the sand phase to the side wall. However, at the same tangential velocity, under the condition of large sand content, the absolute amount of sand particle moving to the side wall is larger. Compared with the monitoring line *L*_MA_, the sand volume fraction in the center and edge of the flow field are both increased. It shows that as the axial distance increases, the sand continues to move towards the side wall of the hydrocyclone under the action of the swirling flow, but the separation of sand is gradually weakened due to the gradual weakening of cyclone separation in the flow field.

The distribution of sand volume fraction on monitoring line *L*_MC_ under different sand content conditions is shown in Fig 21. It can be seen from Fig 21 that the sand volume fraction on monitoring line *L*_MC_ forms a decrease area within the radial range of ±12.5mm, and gradually stabilizes within the radial range of ±12.5mm-±50mm. Among them, because the radius of the separation pipe is 12.5mm, and the position of the monitoring line LMC is close to the top of the separation pipe, the velocity distribution at the monitoring line LMC is affected by the separation pipe which further affects the distribution of sand phase. Therefore, the volume fraction of sand phase fluctuates slightly in the range of ±12.5mm, and a decrease area is formed. In the decrease area, the sand volume fraction at the center of the flow field increases with the increase of sand content. When the sand content is 10% and 40%, the sand volume fraction at the center of the flow field is 4.2% and 25.2% respectively. Compared with the sand volume at the inlet of the hydrocyclone, the sand volume fraction at the center of the flow field under the four sand content conditions decreased by 5.8%, 10.6%, 13.9%, and 14.8%, respectively. It is proved that with the increase of sand content, the amount of sand discharged from the water outlet is larger, but the proportion of sand discharged from the sand outlet is larger.

**Fig 22:**
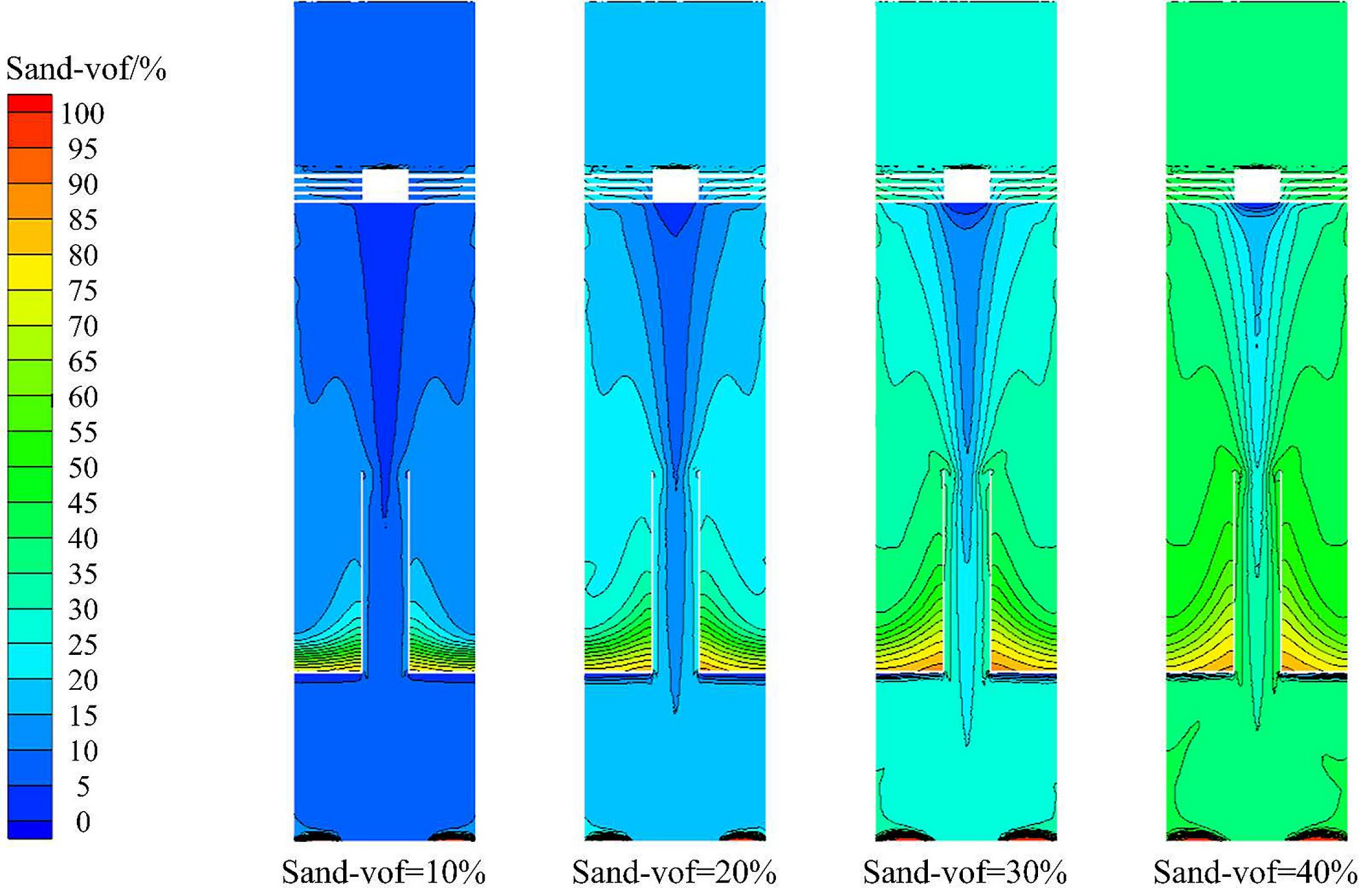
Sand phase distribution in X-Y section under different sand contents.

The sand phase distribution in the X-Y section under different sand content conditions is shown in Fig 22. It can be seen from Fig 13 that the sand phase volume fraction increases from 10% to 40%, and the sand phase volume fraction in the X-Y section increases overall. On the monitoring line *L*_MA_, the sand volume fraction at the radial position of 0mm is all 0. The sand volume fraction increases with the increase of radial position, and the larger the sand content is, the greater the increase of sand volume fraction is. On the monitoring line *L*_MC_, with the increase of sand content, the sand volume fraction increases within the range of ±50mm in the radial position. The surface and curve of sand phase distribution under different sand content conditions show that the law of sand phase distribution is the same.

#### 3.3.4 Flow field characteristics and distribution rules under different sand particle diameter

**Fig 23:**
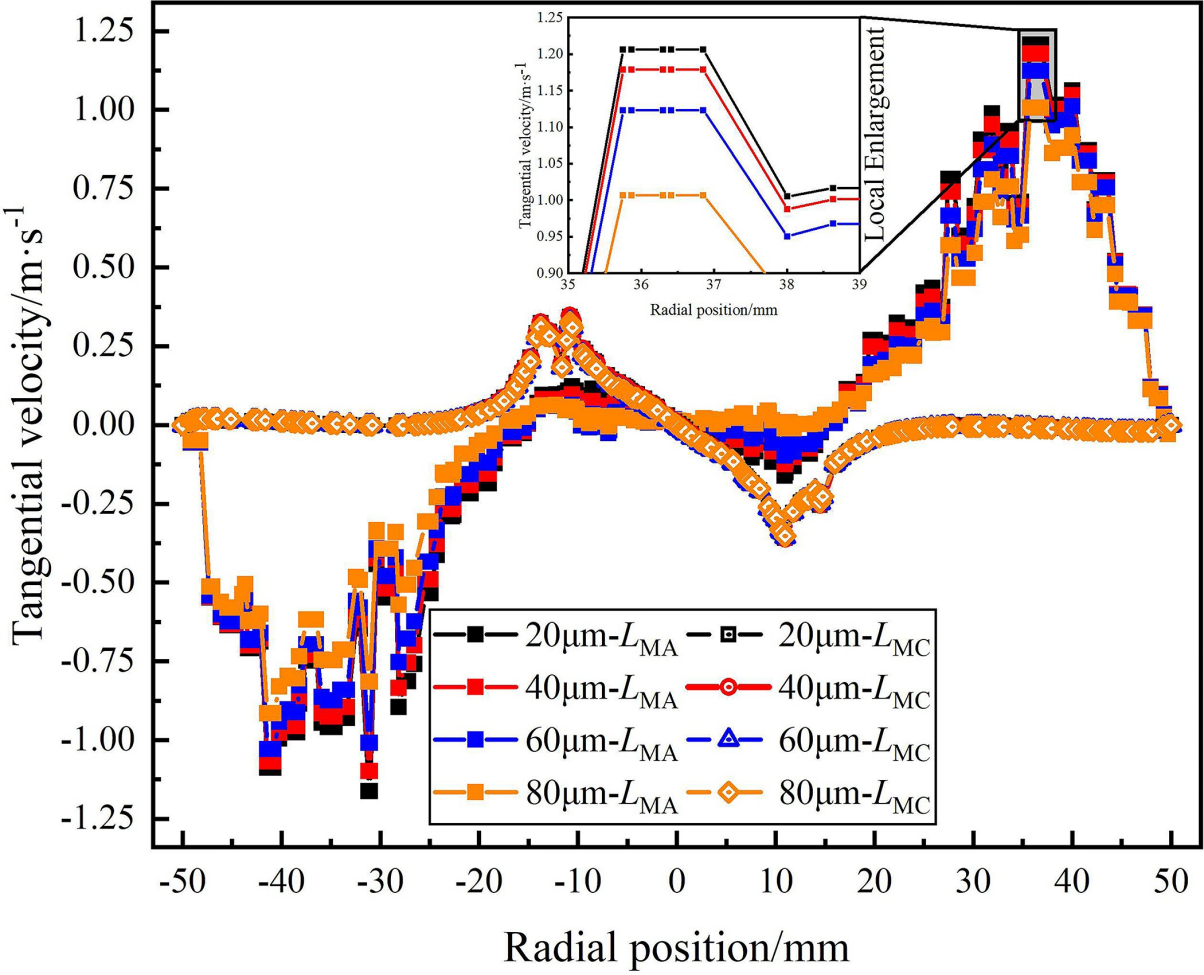
Distribution of tangential velocity on monitoring lines under different sand particle diameter conditions.

Fig 23 shows the distribution of tangential velocity in the swirling flow field under different sand particle diameter. It can be seen from Fig 23 that the maximum tangential velocity on monitoring line *L*_MA_ is at the radial position of ±36mm, and the maximum tangential velocity on monitoring line *L*_MC_ at the radial position of ±12.5mm. And the maximum tangential velocity on monitoring line *L*_MA_ is significantly higher than that on monitoring line *L*_MC_. It shows that as the axial position increases from 121mm to 279mm from the hydrocyclone inlet, the tangential velocity generated by the spiral flow path gradually weakens in the axial and radial directions, and the swirl separation effect in the swirl field also gradually decreases as the tangential velocity decreases. According to Fig 23, the change of sand particle diameter does not have a significant effect on the tangential velocity at the same axial position in the flow field. From the local enlargement, it can be seen that the change of sand particle diameter has only a slight effect on the tangential velocity on monitoring line *L*_MA_. When the sand particle diameter is 20μm, the maximum tangential velocity is 1.21m/s, and when the sand particle diameter is 80μm, the minimum tangential velocity is 1.01m/s. As the sand particle diameter increases from 20 μm to 80 μm, the maximum tangential velocity decreases 0.2m/s. However, on monitoring line *L*_MC_, the maximum tangential velocity in the flow field under different particle diameters is 0.28m/s. It is proved that under the condition of strong swirling flow, the tangential velocity in the flow field decreases with the increase of sand particle diameter, but the change of sand particle diameter has little effect on the tangential velocity.

**Fig 24:**
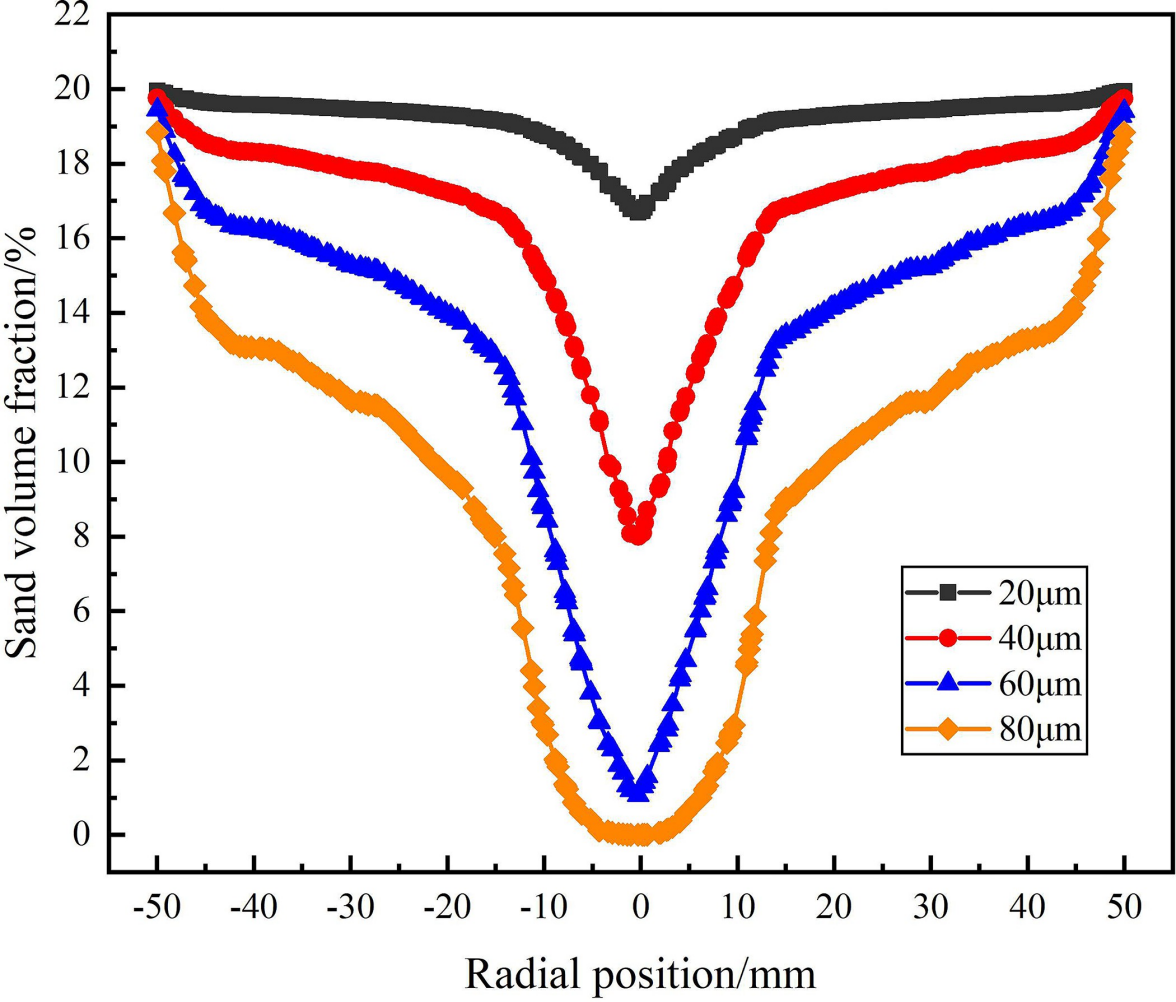
Distribution of sand phase on monitoring line *L*_MA_ under different sand particle diameter conditions.

**Fig 25:**
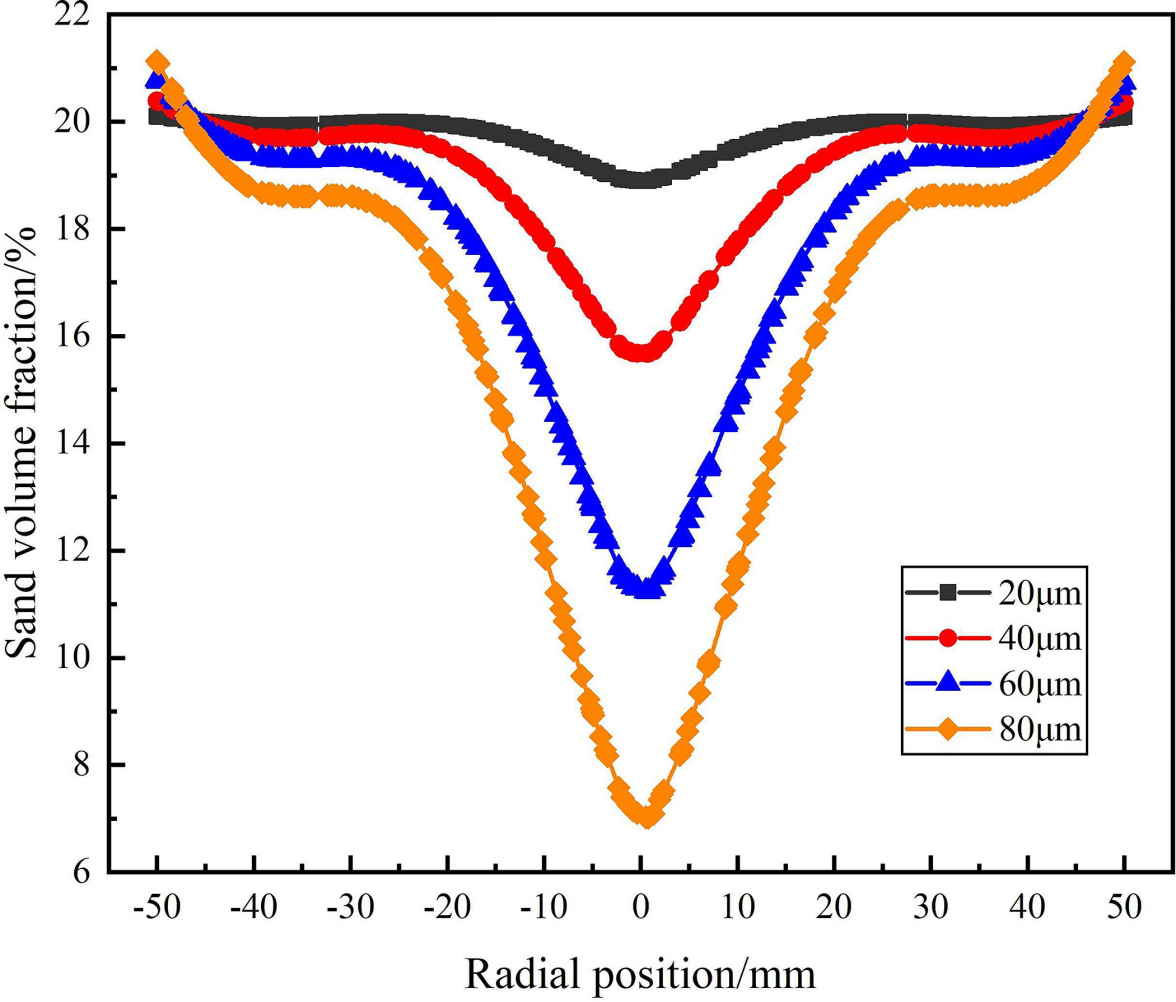
Distribution of sand phase on monitoring line *L*_MB_ under different sand particle diameter conditions.

**Fig 26:**
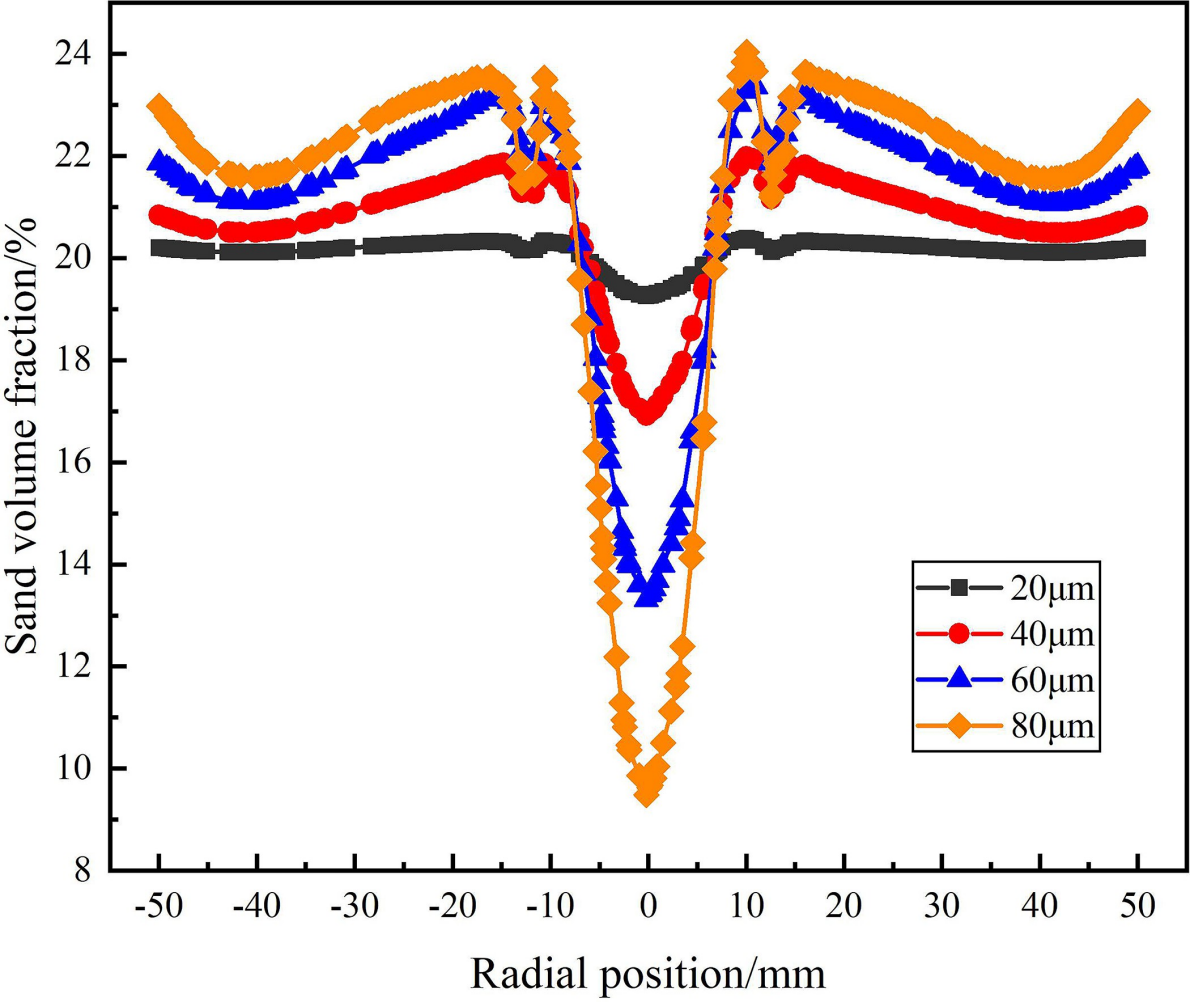
Distribution of sand phase on monitoring line *L*_MC_ under different sand particle diameter conditions.

The distribution of sand phase in the analysis area under different sand particle diameter is shown in Figs 24–26. Among them, Fig 24 shows the distribution of sand volume fraction on monitoring line *L*_MA_. It can be seen from Fig 24 that under the condition of different sand particle diameters, the sand volume fraction shows a distribution law that gradually increases from the center to the edge of the flow field. When the sand particle diameter is in the range of 20–80μm, the sand volume fraction in the swirling field decreases gradually with the increase of particle diameter on monitoring line *L*_MA_, but the sand volume fraction reaches about 20% at the edge of the flow field under different sand particle diameter conditions. At the radial position 0mm, when the sand particle diameter is 20μm and 80μm, the sand volume fraction reaches the maximum and the minimum is 16.9% and 0%, respectively. This is due to the fact that in the circumferential motion, the centrifugal force caused by the increase of sand mass increases, which leads to the more significant the migration of sand particles to the side wall and the smaller the volume fraction of sand phase at the center. It is proved that in the process of cyclone separation, under the condition of the same flow rate and the same sand content, the larger the sand particle diameter is, the easier it is to separate.

The distribution of sand volume fraction on monitoring line *L*_MB_ under different particle diameter conditions is shown in Fig 25. As shown in Fig 25 that the sand volume fraction decreases with the increase of sand particle diameter in the flow field at the distance from the inlet 200mm of the hydrocyclone. Compared with the monitoring line *L*_MA_, the sand volume fraction at the radial position 0mm increases. However, the sand volume fraction is still roughly the same at about 20% at the edge of the flow field under different particle diameters. At this time, the sand volume fraction at the center of the flow field under the four particle diameters is 18.9%, 15.6%, 11.2% and 7%, respectively. Compared with the monitoring line *L*_MA_, which increases by 2%, 7.6%, 9.8% and 7%, respectively. This is due to the increase of the axial distance, the decrease of the tangential velocity in the flow field and the decrease of the centrifugal force on the sand particles, resulting in the weakening of the sand phase separation effect, the weakening of the sand phase migration to the side wall and the accumulation at the center.

Fig 26 shows the distribution of sand volume fraction on monitoring line *L*_MC_ under different sand particle diameter conditions. As can be seen from Fig 26, at the axial position 279mm, the sand volume fraction decreases rapidly in the radial range ±12.5mm and reaches the minimum at 0mm. And the sand volume fraction at the axial center of the flow field is inversely proportional to the sand particle diameter. When the sand particle diameter is 80μm, the sand volume fraction reaches a minimum of 9.4% at the axial center of the flow field. At the same time, due to the effect of the separator pipe wall, there is a small fluctuation in the distribution of sand volume fraction at ±12.5mm. The sand volume fraction near the inner wall of the hydrocyclone is different under the condition of different particle diameters, and the distribution law is positively correlated with the sand particle diameter. When the sand particle diameter is 80μm, the maximum sand volume fraction is 22.9%. When the sand particle diameter is 20μm, the sand volume fraction decreases slightly due to the effect of water phase migration in the range of radial position ±12.5mm, but the overall stability is 20%. This is due to the fact that in the circular motion, the increase of the sand particle size leads to the increase of the centrifugal force, which leads to the result that the larger the sand particle size, the greater the sand volume fraction on the side wall. According to the analysis of the sand volume fraction distribution of from the *L*_MA_ to *L*_MC_ shows that there is a positive correlation between the degree of sand separation and sand particle diameter in the process of cyclone separation. In a certain range, the larger the sand particle diameter, the easier it is to achieve sand phase separation.

### 3.4 Efficiency calculation

**Fig 27:**
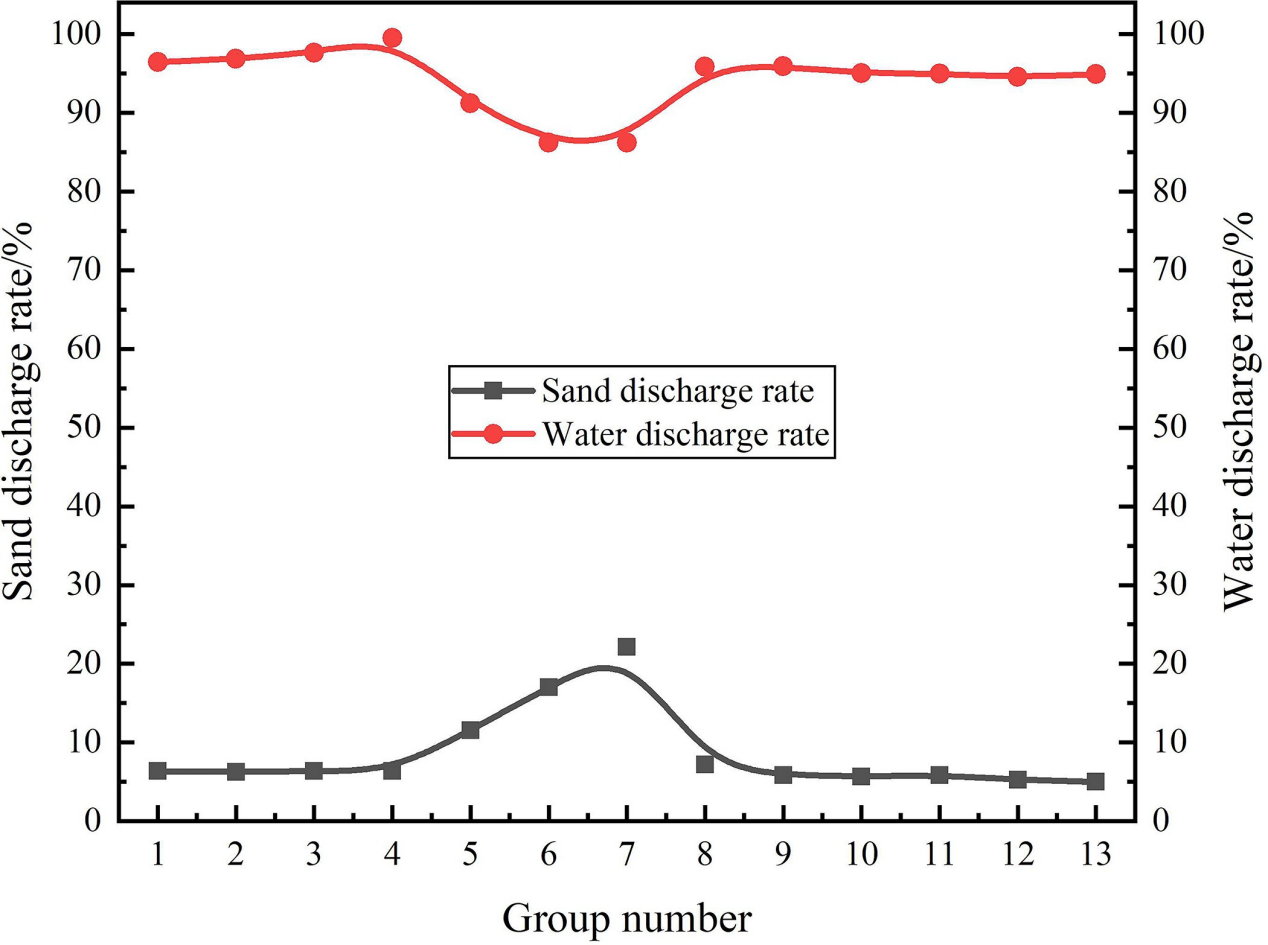
Efficiency calculation.

The sand discharge rate and water discharge rate were calculated according to the Eqs (11)–(12). The *E*_s_ and *E*_w_ calculations for Case1-Case13 are shown in Fig 27. From the distribution law of sand discharge efficiency curve and drainage efficiency curve in Fig 27, it can be seen that there is a negative correlation between sand discharge efficiency and drainage efficiency in hydrocyclone. Among them, the maximum *E*_s_ is 22.1% of Case7, indicating that when the flow rate is 4.83m^3^/h, the sand outlet flow ratio is 20%, the sand content is 20%, and the sand particle diameter is 80μm, the sand phase separation efficiency of this hydrocyclone is the highest. The minimum *E*_w_ is 86.1% of case6 and case7, it indicates that the water discharge efficiency is the same when the flow rate is 4.83m^3^/h, the sand volume fraction is 20%, the sand particle diameter is 80μm, and the sand outlet flow ratio is 15% and 20%, respectively.

## 4. Conclusion

In this paper, based on the hydrocyclone proposed by Chang, which is applicable to the solid fluidization exploitation of natural gas hydrate, the numerical simulation method was used to study the internal flow field characteristics of this type of hydrocyclone under different working conditions and its applicability in the solid fluidization exploitation of natural gas hydrate. Based on the results and discussions above, the following conclusions were drawn:

1. The numerical simulation method was used to study the flow field characteristics and distribution law of the hydrocyclone which is applicable to the solid fluidization exploitation of natural gas hydrate under the conditions of different flow rate, different flow ratio, different sand content and different sand particle diameter. In the same axial position, when the flow rate is in the range of 1.83m^3^/h to 4.83m^3^/h and the sand content is in the range of 10% to 40%, the sand volume fraction decreases at the center of the flow field and increases at the edge of the flow field. When the sand particle diameter increases from 20μm to 80μm, the sand volume fraction decreases at the center of the flow field, but there is no obvious change at the edge of the flow field. When the flow ratio increases from 5% to 20%, there is no significant effect on the distribution of sand volume fraction in the flow field. Therefore, it is suggested that the initial sand removal efficiency of solid fluidization exploitation of natural gas hydrate should be improved by increasing the inlet flow rate in engineering practice.
2. When the axial position in the flow field increases from 121mm to 279mm from the inlet. When the flow rate, flow ratio and sand content are constant, the sand volume fraction increases gradually at the center of the flow field and decreases at the edge of the flow field. When the sand particle diameter is constant, the sand volume fraction has no significant change at the edge of the flow field.
3. The *E*_s_ and *E*_w_ of the hydrocyclone under different working conditions were calculated, and the results show that the maximum *E*_s_ is 22.1% and the minimum *E*_w_ is 86.1% when the flow rate is 4.83m^3^/h, the sand outlet flow ratio is 20%, the sand volume fraction is 20% the sand particle diameter is 80μm. The optimum working conditions for this type of hydrocyclone were obtained. It provides a reference for the practical engineering application of sand pre-separation in solid fluidization exploitation of natural gas hydrate.
4. The hydrocyclone studied in this paper has a low separation efficiency for water and sand phases, and is only applicable to rough pre-separation of sand phase in the process of solid fluidization exploitation of natural gas hydrate, and cannot perform fine separation for complex mixture. In the follow-up research and practical engineering application, the optimization of structural parameters should be further carried out to improve its separation efficiency.

## List of abbreviations

C: Specific heat capacity
d: Separation pipe diameter(mm)
*d*_so_: Sand outlet diameter(mm)
D: Main diameter(mm)
D_ij_: Diffusion term
E: Separation efficiency evaluation value
g: Gravitational acceleration(m/s^2^)
*k*_t_: Fluid thermal conductivity
*l*_i_: Inlet length(mm)
*l*_p_: Separator pipe length(mm)
*l*_s_: Spiral flow path length(mm)
*l*_wo_: Water outlet length(mm)
L: Monitoring lines
m: mass flow rate
*m*_so_: The mass flow rate of sand at sand outlet
*m*_si_: The mass flow rate of sand at inlet
*m*_wi_: The mass flow rate of water at water outlet
*m*_wo_: The mass flow rate of water at inlet
P: Fluid pressure(Pa)
P_ij_: Stress generation term
T: Temperature
u: Velocity(m/s)
Greek letters *σ*: Turbulent Prandtl number
μ: Fluid viscosity
*μ*_*t*_-: viscosity coefficient
ε_ij_: Viscous dissipative term
ρ: Density
$\rho \bar{u_{i}u_{j}}$-: Reynolds stress tensor
Φ_ij_: Pressure strain generating term
*δ*_*ij*_-: Kronecker Symbol
Subscript *i*: Directions
j: Directions
x: position
